# Alpine lichen diversity in an isolated sky island in the Colorado Plateau, USA—Insight from an integrative biodiversity inventory

**DOI:** 10.1002/ece3.7896

**Published:** 2021-07-14

**Authors:** Steven D. Leavitt, Jason Hollinger, Sara Summerhays, Isaac Munger, Jonah Allen, Barb Smith

**Affiliations:** ^1^ M.L. Bean Life Science Museum & Department of Biology Brigham Young University Provo Utah USA; ^2^ Herbarium Department of Biology Western Carolina University Cullowhee North Carolina USA; ^3^ Department of Biology Brigham Young University Provo Utah USA; ^4^ Wildlife Biologist/Botanist, Moab District Manti–La Sal National Forest Moab Utah USA

**Keywords:** ASAP, DNA barcoding, La Sal Mountains, Rocky Mountains, vouchered collections

## Abstract

Lichens are major components of high altitude/latitude ecosystems. However, accurately characterizing their biodiversity is challenging because these regions and habitats are often underexplored, there are numerous poorly known taxonomic groups, and morphological variation in extreme environments can yield conflicting interpretations. Using an iterative taxonomic approach based on over 800 specimens and incorporating both traditional morphology‐based identifications and information from the standard fungal DNA barcoding marker, we compiled a voucher‐based inventory of biodiversity of lichen‐forming fungi in a geographically limited and vulnerable alpine community in an isolated sky island in the Colorado Plateau, USA—the La Sal Mountains. We used the newly proposed Assemble Species by Automatic Partitioning (ASAP) approach to empirically delimit candidate species‐level lineages from family‐level multiple sequence alignments. Specimens comprising DNA‐based candidate species were evaluated using traditional taxonomically diagnostic phenotypic characters to identify specimens to integrative species hypotheses and link these, where possible, to currently described species. Despite the limited alpine habitat (ca. 3,250 ha), we document the most diverse alpine lichen community known to date from the southern Rocky Mountains, with up to 240 candidate species/species‐level lineages of lichen‐forming fungi. 139 species were inferred using integrative taxonomy, plus an additional 52 candidate species within 29 different putative species complexes. Over 68% of sequences could not be assigned to species‐level rank with statistical confidence, corroborating the limited utility of current sequence repositories for species‐level DNA barcoding of lichen‐forming fungi. By integrating vouchered specimens, DNA sequence data, and photographic documentation, we provide an important baseline of lichen‐forming fungal diversity for the limited alpine habitat in the Colorado Plateau. These data provide an important resource for subsequent research in the ecology and evolution of lichens alpine habitats, including DNA barcodes for most putative species/species‐level lineages occurring in the La Sal Mountains, and vouchered collections representing any potentially undescribed species that can be used for future taxonomic studies.

## Graphical abstract

Using an iterative taxonomic approach based on over 800 specimens and incorporating both traditional morphology‐based identifications and information from the standard fungal DNA barcoding marker, we compiled a voucher‐based inventory of biodiversity of lichen‐forming fungi in a geographically limited and vulnerable alpine community in an isolated sky island in the Colorado Plateau, USA—the La Sal Mountains. These data provide an important resource for subsequent lichen biodiversity research in alpine habitats, including DNA barcodes for the vast majority of putative species/species‐level lineages occurring in the La Sal Mountains, crucial information on species distributions, and vouchered collections representing any potentially undescribed species that can be used for future taxonomic studies.

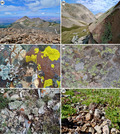

## 1 INTRODUCTION

Accurately and efficiently characterizing species‐level biodiversity is fundamental to investigating a wide range of biological topics, including conservation biology, ecology, evolution, and the impact of climate change (Heywood & Watson, 1995). However, biodiversity assessments are often difficult, particularly for organismal groups with significant proportions of undescribed diversity and in geographic regions that have received limited attention. Sample identification may result in ambiguous specimen assignments using either traditional morphology‐based approaches or DNA sequence‐based approaches (Lücking et al., 2020; Naciri & Linder, 2015). Morphology‐based identifications may not be repeatable, even in cases where identifications are performed by specialists (Carvalho et al., 2011, 2015; Vondrák et al., 2016), and are further confounded by the potential for difficult‐to‐identify specimens, for example, immature or environmentally modified specimens lacking diagnostic features. DNA barcoding represents a transformative and reliable framework for organizing specimens for systematic research and documenting diversity (DeSalle & Goldstein, 2019), in addition to providing genetic data that can be used to infer evolutionary relationships. However, sequence‐based approaches for specimen identification have significant limitations, including incomplete DNA reference libraries, potential to over‐split species‐level lineages, failing to diagnose closely related species, and ambiguous specimen assignments (Leaché et al., 2018; Leavitt et al., 2016; Lücking et al., 2020; Moritz & Cicero, 2004; Naciri & Linder, 2015).

Integrating various lines of evidence in biodiversity inventories, for example, traditional taxonomic approaches with DNA sequence data, can provide improved perspectives into biodiversity surveys (Cao et al., 2016; Sheth et al., 2017). A phylogenetically informed reinterpretation of morphological characters can strengthen taxonomic conclusions and provide direction for nominal taxa in need of revision to more accurately portray evolutionary histories (Hutsemékers et al., 2012). In many groups, presently described species represent only a fraction of the estimated overall diversity (Guiry, 2012; Hawksworth & Lücking, 2017; Pons et al., 2006). Given the limited taxonomic expertise for many groups and the meticulous nature of classical monographic research, integrative approaches can help remedy the current “taxonomic impediment” problem (Dayrat, 2005; Vinarski, 2020) by facilitating the discovery and taxonomic description of novel taxa (Cao et al., 2016).

Alpine, arctic, and Antarctic habitats worldwide support specialized biological communities that are adapted to harsh environmental conditions (Billings, 1974; Chapin & Körner, 1994), and important components of alpine‐adapted communities are often poorly known (Pereira et al., 2012). In these ecosystems, abiotic factors, especially climate, dominate biotic interactions, make them particularly vulnerable to changing climate (Cannone et al., 2007). Detecting changes in occurrence, distribution, or abundance of alpine species is based on knowledge of which species occur in specific locations, information that is conspicuously absent for many organismal groups. In the intermountain region of western North America, expansions and contractions of species’ ranges have proceeded through local movements along elevation gradients to and from scattered high‐elevation patches of habitat throughout the Pleistocene (Guralnick, 2007; Jiménez‐Moreno & Anderson, 2013), including a number of alpine sky islands in the Southern Rocky Mountain Region of the United States. The Southern Rocky Mountains are centered on the ranges of Colorado, extending northwards to the Medicine Bow Mountains in southeast Wyoming and south to the Sangre de Cristo Range in north central New Mexico. It also includes two isolated ranges—the La Sal Mountains in southeastern Utah and the San Francisco Peaks in northern‐central Arizona. Here, isolated alpine habitats are geographically subdivided among different mountain ranges, for example, “sky islands,” harboring unique biodiversity due to a variety of factors spanning multiple spatial and temporal scales (Knowles, 2000; Marx et al., 2017). Climate‐driven distributional shifts lead to complex patterns of diversification and demography in alpine specialists (Galbreath et al., 2009). In the alpine zone of the Southern Rocky Mountains, vascular plants have been systematically inventoried over the past three decades, demonstrating rich plant communities harboring substantial endemism, ca. 10% (Fowler et al., 2014). However, only a limited number of studies investigating lichen diversity in the same region are available (Table 1).

**TABLE 1:** Summary of current understanding of alpine lichen diversity in the Southern Rocky Mountains, USA

Sampling area	# of species	Source
Bald Mountain, Uinta Mountains (Utah)	65	St. Clair et al. (2007)
Beartooth Plateau, Beartooth Mountains (Montana and Wyoming)	80	Eversman (1995)
Mount Audubon, Front Range (Colorado)	86	Egan (1970, 1971)
Niwot Ridge Long‐Term Ecological Research Site, Front Range (Colorado)	92	Flock (1978)
Sierra Blanca Peak, Sierra Blanco Range (New Mexico)	76	Egan (1971)
La Cal Basin, Sangre de Cristo Mountains (New Mexico)	89	Egan (1971)
Lake Peak, Sangre de Cristo Mountains (New Mexico)	76	Egan (1971)
Wheeler Peak, Snake Range (Nevada)	58	Noell and Hollinger (2015)
La Sal Mountains	189+	This study

In many alpine habitats, lichen communities are diverse and found in high relative abundance (Bruun et al., 2006; Imshaug, 1957). Alpine lichens, including those occurring on rock, soil, and/or alpine turf, play important ecological roles, ranging from nutrient cycling to habitat and food sources to soil stabilization (Asplund & Wardle, 2017). Despite the high diversity and abundance of lichens in alpine ecosystems, many of these ecosystems are sensitive to environmental disturbances, including climatic shifts and changes in land management strategies (Cornelissen et al., 2001; Geml et al., 2010; Kranner et al., 2008; St. Clair et al., 2007). Therefore, monitoring alpine lichen communities can provide crucial insight into the biological impacts of climate change in some of the most vulnerable ecosystems. However, the diversity and distributions of many components of alpine lichen communities have been incompletely characterized, emphasizing the need to efficiently generate crucial baseline assessments. Recent, collaborative taxonomic efforts have further highlighted the incredible lichen diversity in high altitude/latitude ecosystems (McCune et al., 2020; Nimis et al., 2018; Spribille et al., 2010, 2020). These studies also reveal that a significant proportion of this diversity has not yet received formal taxonomic recognition. For example, recent lichen diversity inventories in two Alaskan national parks revealed that ca. 10% of the sampled lichens could not be assigned to a known species (Spribille et al., 2010, 2020).

**FIGURE 1:**
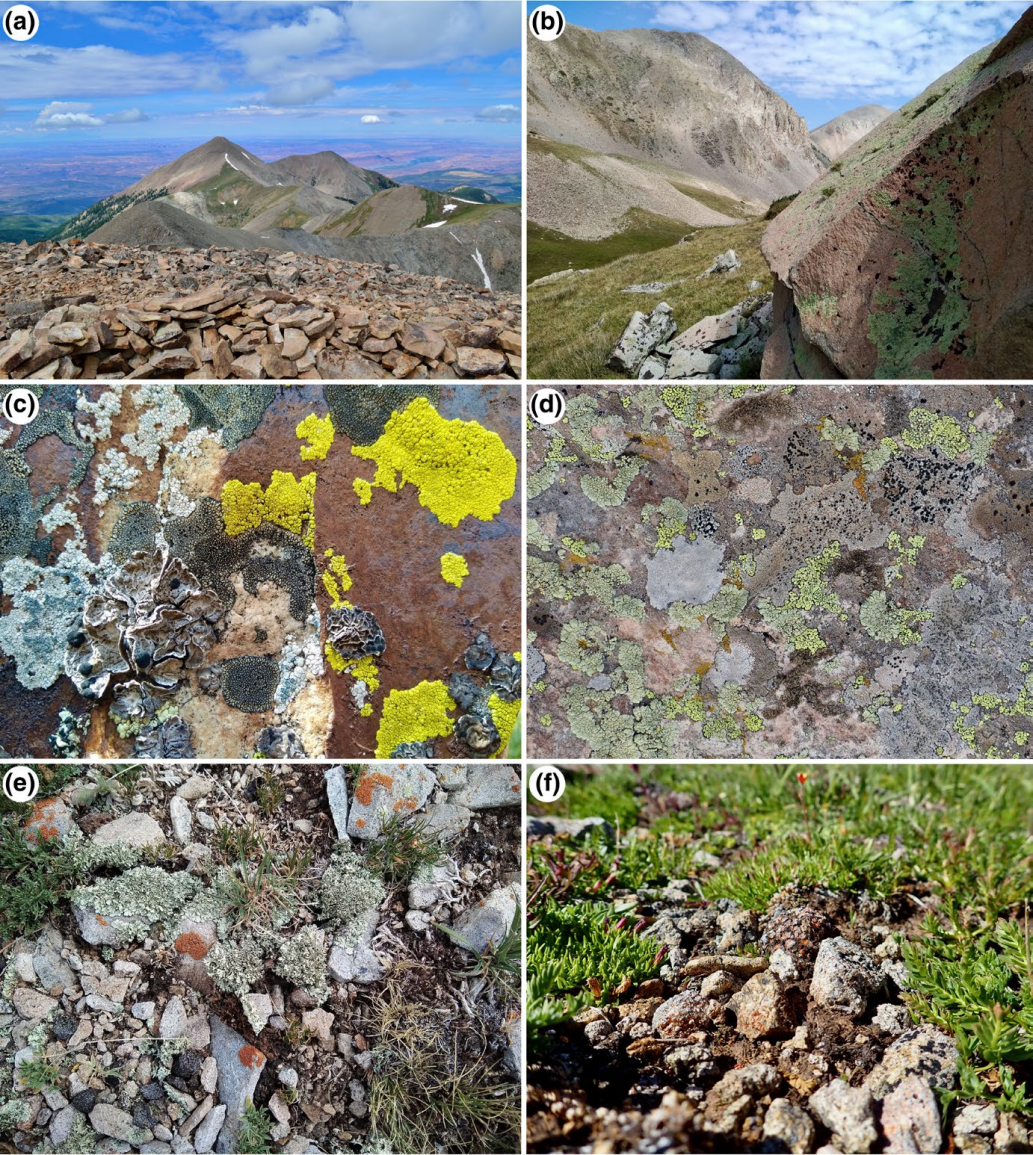
Alpine habitat in the La Sal Range. (a) “Middle Group,” view of Mount Tukuhnikivatz (3,805 m.a.s.l.) from near the summit of Mount Peale (3,879 m.a.s.l.); (b) view from the head of Dark Canyon (3,500 m.a.s.l.), a subalpine basin in the “Middle Group”; (c and d) distinct rock‐dwelling lichen communities; (e and f) distinct soil‐dwelling lichen communities

Effective strategies for using molecular sequence data to aid in the identification of fungi continue to be developed (Abarenkov et al., 2018; Lücking et al., 2020). Coupling these strategies with phenotype‐based data will likely facilitate more effective cataloging the global fungal diversity and provide novel insight into ecological and evolutionary processes (Sattler et al., 2007; Struck et al., 2018). Dispersal of alpine and arctic lichens has received considerable attention in recent years. Frequent long‐distance dispersal has been documented for a number of alpine and arctic lichens (Fernández‐Mendoza & Printzen, 2013; Garrido‐Benavent et al., 2021; Geml et al., 2010; Onuţ‐Brännström et al., 2017) and a “mountain hopping” mechanism of dispersal explains, in part, the broad distribution of many alpine lichens (Garrido‐Benavent & Pérez‐Ortega, 2017). Therefore, the Rocky Mountains in North America play a fundamental role in understanding the processes that influence the distribution of alpine lichen communities (Garrido‐Benavent & Pérez‐Ortega, 2017; Hale et al., 2019; Weber, 2003). To investigate how this might look in practice, here we attempt to characterize lichen‐forming fungal species diversity in the alpine zone of an isolated sky island in the Southern Rocky Mountains using an integrative taxonomic approach—the La Sal Mountains (hereafter the “La Sals”) in the Colorado Plateau in eastern Utah. This insular range is surrounded by semiarid, low‐elevation, canyon dissected terrain (Figure 1), and the high peaks and ridgelines of the La Sals support one of the few true alpine lichen communities in the Colorado Plateau, including ca. 320 ha of vegetated alpine turf. The alpine habitat is known to support a number of sensitive vascular plants, including the endemic La Sal daisy (*Erigeron mancus* Rydb.; Fowler & Smith, 2010). Limited, informal surveys in alpine habitats in the La Sals have suggested the potential for a robust alpine lichen community. However, the origin and establishment of this alpine lichen community is not currently well understood for this unique ecosystem in the Colorado Plateau. Furthermore, specific responses of lichens to ongoing climate change and changes in land management strategies in the alpine habitat in the La Sals remain unknown. Crucial insight might be gained into these processes by more fully characterizing the alpine lichen community in the La Sals.

For this study, our aim was to characterize lichen‐forming fungal diversity occurring in alpine habitat on the insular La Sal Mountains in the Colorado Plateau. We ask (a) how might incorporating DNA sequence data in general surveys influence our perspective of species‐level diversity? (b) Can these data expedite identifying challenging species complexes that require additional taxonomic work? And (c) how well do current DNA sequence repositories used for DNA barcoding reflect actual diversity? To address these questions, we used an integrative taxonomic approach based on recently collected vouchered specimens, incorporating both traditional morphology‐based identifications and information from the standard fungal DNA barcoding. Our findings provide a baseline for future studies investigating ecology and evolution in alpine lichens in western North America.

## 2 MATERIALS AND METHODS

### 2.1 Study site, sampling, and morphology‐based identifications

The La Sals comprise three distinct groups—the “North,” “Middle,” and “South” groups (File S1), which were formed in the Laramide orogeny during the Oligocene when intrusive, dioritic magmas uplifted overlying sedimentary rocks (Hunt & Waters, 1958; Nelson et al., 1992). Our sampling focused exclusively on habitats above timberline in the “North” and “Middle” groups—ca. 3,250 ha above 3,350 m above sea level (m.a.s.l.). Sites were selected to represent the range of geological and ecological features found in these alpine habitats (Figure 1; File S1). Most of the alpine habitat is dominated rock/talus, with only 320 ha of vegetated alpine habitat. In addition to alpine peaks and ridgelines, we collected in two alpine basins, Beaver Basin in the “North Group” and Dark Canyon in the “Middle Group” (Figure 1b). We used an opportunistic taxonomic sampling approach, for example, “intuitive meander” (Whiteaker et al., 1998), aiming to generate a comprehensive but qualitative overview of diversity in the alpine region of the La Sals. Vouchered collections were made during the summers of 2018 and 2019 on all available substrates, including soil, mosses, vascular plants (including limited lignum found above the current timberline), and rock surfaces. Lichens from timberline krummholz—stunted conifers occurring near tree line—were not sampled. Visual assessments of lichens were made in the field with a 10× hand lens, and representatives of the range of observed variation were collected and returned to the laboratory for indentification. If specimens were observed representing lichens that we had collected previously, they were not necessarily collected at additional sites.

Lichen specimens collected during the fieldwork phase of this project are deposited in the Herbarium of Non‐Vascular Cryptogams (BRY‐C) at Brigham Young University, Provo, Utah, USA. Photographs of new collections were made using an Olympus DP‐22 camera attached to an Olympus SZH‐10 stereomicroscope. Compilations of stacked images were done with Zerene Stacker 1.04 (Richland, Washington, USA). We used the Consortium of North American Lichen Herbaria (https://lichenportal.org) to identify historic lichen records representing specimens from the alpine regions of the La Sals.

### 2.2 DNA extraction, amplification, and sequencing

We attempted to generate molecular sequence data for the mycobiont from all vouchered collections made in 2018/2019. Many vouchers included additional, accessory lichens; and efforts were made to sample material from these accessory lichens, along with the targeted lichen. From selected specimens, a small portion of the thallus free of visible contamination was excised, and total genomic DNA was extracted using the Wizard Genomic DNA Purification Kit (Promega, USA). We amplified the fungal internal transcribed spacer region (ITS) using primers ITS1 (Gardes & Bruns, 1993) with ITS4 (White et al., 1990). Polymerase chain reaction (PCR) amplifications were performed using Ready‐To‐Go PCR Beads (GE Healthcare, Pittsburgh, PA, United States), with cycling parameters following a 66–56°C touchdown reaction (Lindblom & Ekman, 2006). PCR products were visualized on 1% agarose gel and enzymatically cleaned using ExoSAP‐IT Express (USB, Cleveland, OH, United States). Complementary strands were sequenced using the same primers used for amplifications, and sequencing reactions were performed using BigDye 3.1 (Applied Biosystems, Foster City, CA, United States). Products were run on an ABI 3730 automated sequencer (Applied Biosystems) at the DNA Sequencing Center at Brigham Young University, Provo, UT, United States. In cases where the initial PCR and/or sequencing reactions failed to yield high‐quality reads or resulted in unexpected/questionable sequences, PCR and sequencing reactions were attempted up to three times per problematic sample.

### 2.3 DNA‐based inference of mycobiont candidate species and phylogenetic reconstructions

All sequences generated for the study were subjected to an initial “blastn” search against GenBank's nucleotide collection (Altschul et al., 1990) to confirm family‐level membership. Exploratory multiple sequence alignments (MSAs) of all ITS sequences generated for this study resulted in unreliable alignments due to the high variability of the ITS region at deeper fungal taxonomic levels. Therefore, all subsequent MSAs generated here were performed at the mycobiont family‐level. Family‐level MSAs were generated using the program MAFFT v7 (Katoh & Toh, 2008; Rozewicki et al., 2017). We implemented the G‐INS‐i alignment algorithm and “1PAM/K = 2” scoring matrix, with an offset value of 0.1, the “unalignlevel” = 0.4, and the remaining parameters were set to default values.

To circumscribe candidate mycobiont species from the family‐level ITS MSAs, we used Assemble Species by Automatic Partitioning (ASAP; Puillandre et al.). ASAP is a recently developed method that circumscribes species partitions using an implementation of a hierarchal clustering algorithm based on pairwise genetic distances from single‐locus sequence alignments (Puillandre et al., 2021). The pairwise genetic distances are used to build a list of partitions ranked by a score, which is computed using the probabilities of groups to be panmictic species. ASAP, therefore, provides an objective approach to circumscribe relevant species hypotheses as a first step in the process of integrative taxonomy.

Each family‐level ITS MSA was analyzed under a maximum likelihood (ML) criterion as implemented in IQ‐TREE v2 (Nguyen et al., 2014), with 1,000 ultrafast bootstrap replicates (Hoang et al., 2017), with the best‐fitting substitution model for the entire ITS region selected using ModelFinder (Kalyaanamoorthy et al., 2017). Trees were visualized using FigTree v1.4.4 (Rambaut, 2008). Species partitions inferred from the family‐level ASAP analyses were compared to phylogenetic reconstructions to determine reasonable DNA‐based candidate species (DNA‐CS) using the criterion of reciprocal monophyly in DNA‐CSs, in addition to qualitative assessments of branch lengths and lichen morphology (see below).

### 2.4 Integrative taxonomy

Incorporating phenotypic data in the assessment of DNA‐CSs through integrative taxonomy provides critical information for evaluating species‐level diversity in lichen‐forming fungi (Lücking et al., 2020). Phenotypic traits of all vouchered specimens were examined in light of the DNA‐CSs delimited using ASAP and the phylogenetic inferences. Diagnostic features from relevant taxonomic keys and monographs, as well as a variety of online resources, were considered. As needed, thin‐layer chromatography (Culberson, 1969; Orange et al., 2001) was used to aid with specimen identifications.

To characterize how DNA‐CSs compared with phenotypically circumscribed species and currently available sequence data in GenBank, each DNA‐CS was categorized within the following categories: “match”—ITS sequences ≥98% similar to sequences from the same taxon currently available in GenBank; “species complex”—ITS sequences ≥97% similar to morphologically similar species but represented by multiple candidate species; “affinity”—sequences representing candidate species not recovered as monophyletic, represented by multiple candidate species; “mismatch”—ITS sequences <95% similar to sequences from the same taxon currently available in GenBank; and “no comparison”—sequences from identified species were not available in GenBank. We note that currently available ITS sequence data in GenBank represents only a small portion of extant fungal species, and many of the sequences are incorrectly identified to species level (Nilsson et al., 2006). Furthermore, pairwise similarity‐based approaches, such as BLAST, can provide misleading perspectives into taxonomic assignment and relationships (Lücking et al., 2020). Hence, we used the Protax‐fungi pipeline for taxonomic placement using ITS sequences, as implemented on the PlutoF platform and using the UNITE database (Abarenkov et al., 2010, 2018). Protax‐fungi provides statistical assessment of taxonomic assignment precision, from species to phylum ranks, here choosing a plausible classification value of 0.05.

## 3 RESULTS

From a total of 446 vouchered collections housed at BRY‐C, DNA was extracted from 805 distinct lichen thalli (many vouchers included multiple, distinct lichens), and we successfully amplified ITS sequence data from 734 thalli (Table S1; GenBank accession numbers MZ243469–MZ244202). All supplementary files, alignments, and photographs of sampled thalli are available in Dryad: https://doi.org/10.5061/dryad.9ghx3ffh4.

### 3.1 Genetic diversity of lichen‐forming fungi and DNA‐based species delimitation

A total of 24 families of lichen‐forming fungi were represented by ITS sequence data, with the most frequently collected families including the following: Lecanoraceae (*n =* 238), Physciaceae (*n* = 71), Megasporaceae (*n* = 67), Teloschistaceae (*n* = 66), and Candelariaceae (*n* = 57) (Table S1). The family‐level ASAP species delimitation analyses resulted in a total of 244 species partitions, with an average of 2.8 sequences per species partition and ranging from one to seven sequences per species partition (Table 2). High genetic diversity was observed in many traditional, phenotype‐based species, often with multiple ASAP species partitions inferred from nominal species, and these putative species complexes represented 96 of 244 ASAP species partitions (Appendix S1). Family‐level phylogenetic reconstructions also revealed high genetic diversity; and, in general, ASAP species partitions corresponded with distinct, reciprocally monophyletic clades (File S3). Comparing ASAP species partitions with family‐level phylogenetic reconstructions reduced the DNA‐CSs from 244 to 222 (Appendix S1; File S3), combining several closely related and/or nonmonophyletic ASAP species partitions, particularly in the families Candelariaceae, Lecanoraceae, and Teloschistaceae (Table 2).

**TABLE 2:** Summary of lichen‐forming fungal species diversity collected in the La Sal Mountains in eastern Utah, USA

Family	# ASAP species	Candidate species	Integrative species (species complexes)
Acarosporaceae (16)	10 (1.6)	12	11
Caliciaceae (6)	3 (2)	3	3
Candelariaceae (57)	23 (2.5)	18	8
Cladoniaceae (24)	9 (2.6)	9	5
Collemataceae (2)	2 (1)	2	2
Gyalectaceae (1)	1 (1)	1	1
Lecanoraceae (238)	76 (3.3)	69	43
Lecideaceae (2)	1 (2)	1	1
Megasporaceae (67)	18 (3.7)	22	14
Parmeliaceae (22)	3 (7)	5	5
Peltigeraceae (19)	6 (3.2)	6	6
Pertusariaceae (3)	1 (3.0)	1	1
Physciaceae (71)	17 (4.2)	18	18
Psoraceae (21)	3 (7)	3	3
“Pseudoaspicilia” (1*)	1 (1)	1	1
Ramalinaceae (1*)	1(1)	1	1
Rhizocarpaceae (36)	6 (6)	6	6
Sporastatiaceae (7)	4 (1.75)	1	1
Stereocaulaceae (10)	4 (2.5)	4	4
Teloschistaceae (66)	26 (2.5)	19	19
Tephromelataceae (12)	2 (6)	2	1
Thelotremataceae (1)	1 (1)	1	1
Umbilicariaceae (17)	15 (1.4)	4	4
Verrucariaceae (38)	11 (3.5)	13	12
Species from recent collections w/o sequence data	NA	5	5
Lichenicolous fungi w/o sequence data	NA	3	3
Additional species from historic collections	NA	10	10
24 families	244 total ASAP species (2.8)	240 total candidate species	189 total integrative species

Note

The first column lists the family and number of sampled thalli represented by ITS sequence data in parentheses; the second column reports the number of species (average number of sequences/species) inferred using the newly proposed Assemble Species by Automatic Partitioning (ASAP) approach to empirically delimit candidate species‐level lineages from family‐level multiple sequence alignments; the third column reports the number of candidate species based on combining information from the ASAP partitions and phylogenetic reconstructions; and the fourth column reports integrative species, combining information from the DNA‐based candidate species with morphological data.

### 3.2 Integrative taxonomy

Our integrative specimen identification approach (phenotype + sequence data) resulted in a total of 189 species (Table 2). In many cases, morphologically similar specimens were recovered in divergent, well‐supported phylogenetic lineages; and from an integrative perspective, these were combined into a single species (e.g., taxa in Candelariaceae and Megasporaceae; File S3). In other cases, traditionally accepted species known to display considerable morphological variable were recovered in divergent, well‐supported phylogenetic lineages, and these were also combined into a single “integrative” species.

Over half (54%) of all candidate species inferred in this study were ≥98% similar to sequences representing the same taxa and presently available on GenBank (searched 15 December 2020). Over a third of all candidate species appear to belong to species complexes of morphologically similar taxa with at least 97% genetic similarity, representing 29 putative species complexes (Appendix S1; File S3). In contrast, nearly a third (31%) of all candidate species had no match on GenBank (sequences from identified species not presently available in GenBank) or their sequences from identified species were highly dissimilar from sequences from the same taxon on GenBank. Of the newly generated sequences that were <95% similar to sequences presently available in public databases, most belonged to members of Lecanoraceae, Megasporaceae, Psoraceae, Rhizocarpaceae, and Verrucariaceae (Table S1). Approximately 5% of new sequences were <90% similar sequences presently available on GenBank. In the Protax‐fungi analysis, over 68% of sequences could not be assigned to species‐level rank with statistical confidence, and nearly 8% of sequences were not assigned any taxonomic rank (File S4).

### 3.3 Historic collections and taxa lacking DNA sequencing data

Ten lichens collected in alpine regions of the La Sals between 1954 through the 1980s were not observed during the 2018/2019 fieldwork, including two conspicuous and notable macrolichens, *Brodoa oroarctica* (Krog) Goward and *Hypogymnia austerodes* (Nyl.) Räsänen (Appendix S1). We failed to obtain DNA sequence data from a limited number of potentially unique lichens observed during the 2018/2019 fieldwork. A single specimen representing *Rhizocarpon disporum* (Nägeli ex Hepp) Müll. Arg. was photographed above Beaver Basin in 2019 but not collected. Additional species collected for which all sequencing attempts failed include the following: *Athallia saxifragarum* (Poelt) Arup, Frödén & Søchting (*Leavitt 18‐651*), *Buellia* De Not. sp. (*Leavitt 18‐426*), *Rinodina imshaugii* Sheard (*Leavitt 18‐574*), and *R*. *olivaceobrunnea* C. W. Dodge & Baker (*Leavitt 18‐626*). Similarly, sequencing efforts for a number of cyanobacteria‐associated lichens, largely in Collemataceae, frequently resulted in unusable chromatograms. Finally, six lichenicolous fungi were documented but are not represented by DNA sequence data (Appendix S1).

## 4 DISCUSSION

Despite the limited alpine habitat on the insular La Sals in the Colorado Plateau, USA, we document the most diverse alpine lichen communities known to date from the southern Rocky Mountains (Table 1), with at least 189 documented species of lichen‐forming fungi (Appendix S1). The actual number of species in the alpine habitat in the La Sals is likely higher. Our integrative data (DNA sequence data + phenotype‐based inference) suggest that a substantial number of nominal lichens occurring in the La Sals are comprised of multiple candidate species‐level lineages (DNA‐CS; Appendix S1; File S3). Including DNA‐CS within these species complexes leads to a greater than 25% increase in species‐level diversity, with at least 52 additional candidate species (Appendix S1). Furthermore, additional surveys, including alternative sampling strategies, would likely result in additional species not documented here (e.g., Vondrák et al., 2016). A preliminary checklist, with accompanying taxonomic notes, is reported in companion paper (Leavitt et al., in preparation), and below, we discuss the implications of our integrative inventory approach in better understanding alpine lichen diversity.

Despite the important perspective gained from integrative inventories, our results highlight several potential challenges that may continue to impede inventories of understudied organisms. While our study captured some of the highest levels of alpine lichen diversity in the western continental United States, the combination of traditional voucher‐based approaches with DNA barcode sequencing individual lichen thalli was relatively labor‐ and cost‐intensive. Furthermore, the taxonomic status for a significant proportion of this diversity remains ambiguous despite integrating morphological features with sequence data. Subtle or difficult to discern diagnostic traits, conflicting interpretations of morphological variation, including the recognition of morphologically cryptic species‐level lineages, and phenotypic convergence may potentially confound inferences from taxonomic inventories (Argüello et al., 2007; Crespo & Pérez‐Ortega, 2009; Printzen, 2009). Accurate and precise identifications of lichen fungi from biodiversity inventories is often labor‐intensive, often requiring a team of taxonomic experts to appropriately interpret variation (Lücking et al., 2020; McCune et al., 2020; Spribille et al., 2020).

Similarly, DNA barcoding approaches are confounded by inherent limitations with the standard fungal DNA barcoding marker, the ITS, coupled with the lack of comprehensive DNA reference libraries for effective taxonomic assignment (Lücking et al., 2020; Nilsson et al., 2019). While the standard barcode marker for fungi can diagnose distinct species‐level lineages in many cases (Schoch et al., 2012), variation in rDNA can in some species complexes provides biased perspectives, potentially over‐splitting natural species‐level groups. Intraspecific and intragenomic variation in this repeat region is not well known in fungi (Lofgren et al., 2019). In some lichen‐forming fungi, for example, the *Rhizoplaca melanophthalma* aggregate, minimal intragenomic variation was observed (Bradshaw et al., 2020) and the ITS region successfully diagnoses the majority of species in this complex (Leavitt et al., 2013). However, based on genomic data from members of the *R*. *melanophthalma* aggregate, some highly divergent ITS sequences inferred as distinct candidate species in single‐locus species delimitation analyses likely belong to a single species (Bradshaw et al., 2020; Keuler et al., 2020). Inferences from single‐locus species delimitation analyses, such as those performed in this study, are inherently limited (Fujita et al., 2012), and most DNA‐based species hypotheses will likely need to be investigated on a case‐by‐case basis.

Limitations with currently available reference libraries for sequence comparison were manifested in the low proportion successful taxonomic assignment of DNA‐CS at the species level (File S4), with over 68% of sequences that could not be assigned to species‐level rank with statistical confidence. Confounding the poor success in DNA‐based taxonomic assignment, nearly a third of all morphologically identified species were not represented by sequence data in public repositories, and in other cases, sequence data from unidentifiable alpine lichens did not closely match to currently available sequence data (Appendix S1). The results support the perspective that substantially increasing the number of sequences based on verified material will be essential for efficient DNA barcode identification (Lücking et al., 2020; Nilsson et al., 2019). In general, for biodiversity surveys where comprehensive taxonomic treatments are impractical, best practices for reporting uncertain identifications are not well established. However, with recent improvements in how unclassifiable species hypotheses are handled in the UNITE database, these “dark” taxa can now be integrated with the taxonomic backbone of the Global Biodiversity Information Facility and an unlimited number of parallel taxonomic classification systems are supported (Nilsson et al., 2019). Without high‐quality sequence databases that are thoroughly curated by taxonomists and systematists, integrative biodiversity inventories of lichen‐forming fungi will remain labor‐ and cost‐intensive (Begerow et al., 2010).

The results of this study expand novel sequence data into publicly available repositories, providing the first ITS sequences for many species and candidate species‐level lineages. Furthermore, we link these sequence data to digital imagery from vouchered specimens (File S1), in addition to the physical vouchered collections (Appendix S1). We documented unexpected ITS sequence variation in multiple nominal species occurring in the La Sals (Appendix S1; File S3), and the species complexes inferred here provide important direction for identifying lineages that require additional research. By providing publicly available ITS sequence data for the majority of specimens collected for this study, our results can be easily integrated in future research using the formal barcoding marker for fungi (Schoch et al., 2012). Rather than relying exclusively on phenotype‐based identifications that may be biased in comparison with other studies (Brunialti et al., 2012, 2019; Giordani et al., 2009), these ITS data can be directly integrated into a wide range of future studies using the standard DNA barcoding marker for fungi. Our molecular‐based approach for initial species delimitation using ASAP provided only an initial perspective into diversity, providing important direction for future taxonomic research. With ongoing taxonomic revisions of lichen‐forming fungi, including the description of new taxa, the sequence data generated here will facilitate more accurate reassignment of specimens from the La Sals to the appropriate taxonomic group.

While the high levels of diversity documented in this study are striking, we predict that other alpine habits in the southern Rocky Mountains may have similar levels of diversity. Small crustose lichens on rocks and soil are common in most alpine regions but are easily overlooked in vegetation surveys (Ahti & Oksanen, 1990). Even when recognized, these crustose lichens may not be documented because of difficulties with identification due to a lack of diagnostic features or environmental modifications in extreme habitats (Kappen, 1973; McMullin et al., 2020). Here, we aimed to overcome these challenges by integrating vouchered specimens (permanently housed in BRY‐C), photographic documentation (File S2), and DNA sequence data (deposited in GenBank) to create a robust, transparent inventory of lichen‐forming fungal diversity for the limited and vulnerable alpine habitat in the Colorado Plateau, USA. These data provide an important resource for subsequent biodiversity research in alpine habitats, including DNA barcodes for most putative species occurring in the La Sals, crucial information on species distributions, and vouchered collections representing any potentially undescribed species that can be used for phenotypic comparisons.

The opportunistic sampling approach implemented in this study was intended to capture the broad range of species diversity in alpine habitat on the La Sals, rather than provide quantitative insight into distribution patterns or site‐specific species richness (McCune & Lesica, 1992; McMullin et al., 2010; Newmaster et al., 2005). Although different habitats throughout the alpine zone in the La Sals, for example, talus slopes, alpine turf, and late snowmelt areas, support distinct lichen communities, we cannot make robust comparisons among these different communities given the limitations with present sampling. Future ecological sampling approaches will be essential to characterizing factors influencing lichen community assembly and monitoring disturbances and ecological changes. For example, mountain goats (*Oreamnos americanus*) were released in 2013 in the La Sal Mountains with notable, site‐specific impact on alpine communities, including lichens (Leavitt & Smith, 2020). The long‐term impact of the large ungulates on alpine communities will require long‐term monitoring. Coupling environmental sampling approaches with the DNA reference sequences provided here will facilitate more efficient sampling strategies for DNA‐based monitoring of ecological changes in alpine lichen communities in the La Sals. Both amplicon‐based and whole‐genome shotgun metagenomic approaches using environmental samples have been shown to capture higher levels of diversity than traditional inventory strategies (Keepers et al., 2019; Wright et al., 2019), the trade‐offs among cost, speed, accuracy, and precision of metagenomic approaches must be carefully considered (Lücking et al., 2020). Metagenomic approaches may also reflect the dispersal of propagules of lichen fungi and not necessarily mature, established lichens, inflating species richness (Keepers et al., 2019; Tripp et al., 2019). Incorporating field observations, vouchered specimens, and phenotype‐based data is essential for utilizing metagenomic methods for characterizing fungal diversity.

The origin and stability of members of the alpine lichen community in the isolated La Sal Range remain in question. With limited alpine habitat in the La Sals, major climatic fluctuations during the Pleistocene and geographic distance from other more extensive alpine habitats, one might predict that fluctuating conditions might lead to depauperate alpine lichen communities (Jiménez‐Moreno & Anderson, 2013; Louderback et al., 2015). However, we observed the opposite pattern—species‐rich and genetically diverse alpine lichen communities in the La Sals (Appendix S1 and File S3). High levels of intraspecific genetic diversity were common across most families of lichen‐forming fungi occurring in the La Sals (File S3), often with nominal species represented by multiple DNA‐CSs. These results indicate that a large number of lichens were able to successfully disperse, establish, and persist in the isolated alpine habitat in Colorado Plateau. Did members of these communities persist in situ in refugia (Holderegger & Thiel‐Egenter, 2009)? The steep‐sloped ridgelines and conical, eroded peaks in the La Sals may have existed as nunataks (Richmond, 1962), providing suitable habitat for long‐term persistence even during Pleistocene glacial cycles and resulting in the high diversity observed in this study. Contemporary climate change is now having cascading effects on ecosystems, affecting community structure, biotic interactions, and biogeochemistry (Abbott & Jones, 2015; Ernakovich et al., 2014). These sky islands offer considerable potential for investigating not only how different evolutionary and ecological processes structure biological communities but also the impact of modern habitat changes (Czortek et al., 2018). What is the role and frequency of contemporary or recent dispersal events in driving alpine lichen community structure? Similarly, what are the roles of environmental filters and other historical and stochastic factors driving lichen community structure in alpine sky islands (Marx et al., 2017)? In contrast to other alpine regions in the Rocky Mountains where macrolichens comprise an important component of alpine lichen communities (Imshaug, 1957), alpine‐specific macrolichens, for example, cetrarioid species, *Thamnolia subuliformis* (Ehrh.) W. L. Culb., etc., were notably absent from alpine habitats in the La Sals, except for a single observation of *Evernia divaricata* (L.) Ach. Our hope is that results from this study will provide further impetus to explore questions relating to the origin and stability of alpine lichen communities.

## CONFLICT OF INTEREST

The authors declare no conflicting interests.

## AUTHOR CONTRIBUTIONS

**Steven D. Leavitt:** Conceptualization (lead); Data curation (lead); Formal analysis (lead); Funding acquisition (lead); Investigation (lead); Methodology (lead); Project administration (lead); Resources (lead); Software (lead); Supervision (lead); Validation (lead); Visualization (lead); Writing‐original draft (lead); Writing‐review & editing (lead). **Jason Hollinger:** Conceptualization (supporting); Data curation (supporting); Formal analysis (supporting); Investigation (supporting); Methodology (supporting); Validation (supporting); Writing‐review & editing (supporting). **Sara Summerhays:** Data curation (supporting); Investigation (supporting); Software (supporting); Visualization (supporting). **Isaac Munger:** Data curation (supporting); Methodology (supporting); Visualization (supporting). **Jonah Allen:** Methodology (supporting); Visualization (supporting). **Barb Smith:** Conceptualization (supporting); Funding acquisition (equal); Methodology (supporting); Resources (supporting); Supervision (supporting); Writing‐review & editing (supporting).

## Supporting information

File S1

File S2

File S3

File S4

Table S1

Appendix S1

## Data Availability

DNA sequences: GenBank accessions Nos. MZ243469–MZ244202. Final family‐level multiple sequence alignments and topologies; photographs of sampled specimens; and supplementary files: Dryad https://doi.org/10.5061/dryad.mpg4f4r08.
