# Supplementary material for: Alpine lichen diversity in an isolated sky island in the Colorado Plateau, USA—Insight from an integrative biodiversity inventory
Source: Ecol Evol. 2021 Jul 14;11(16):11090–101. doi: 10.1002/ece3.7896 (PMC8366874; doi:10.1002/ece3.7896)

Satellite view of Colorado Plateau in western North America, with the La Sal Range in southeastern Utah outlined. Thickened lines indicate state boundaries.

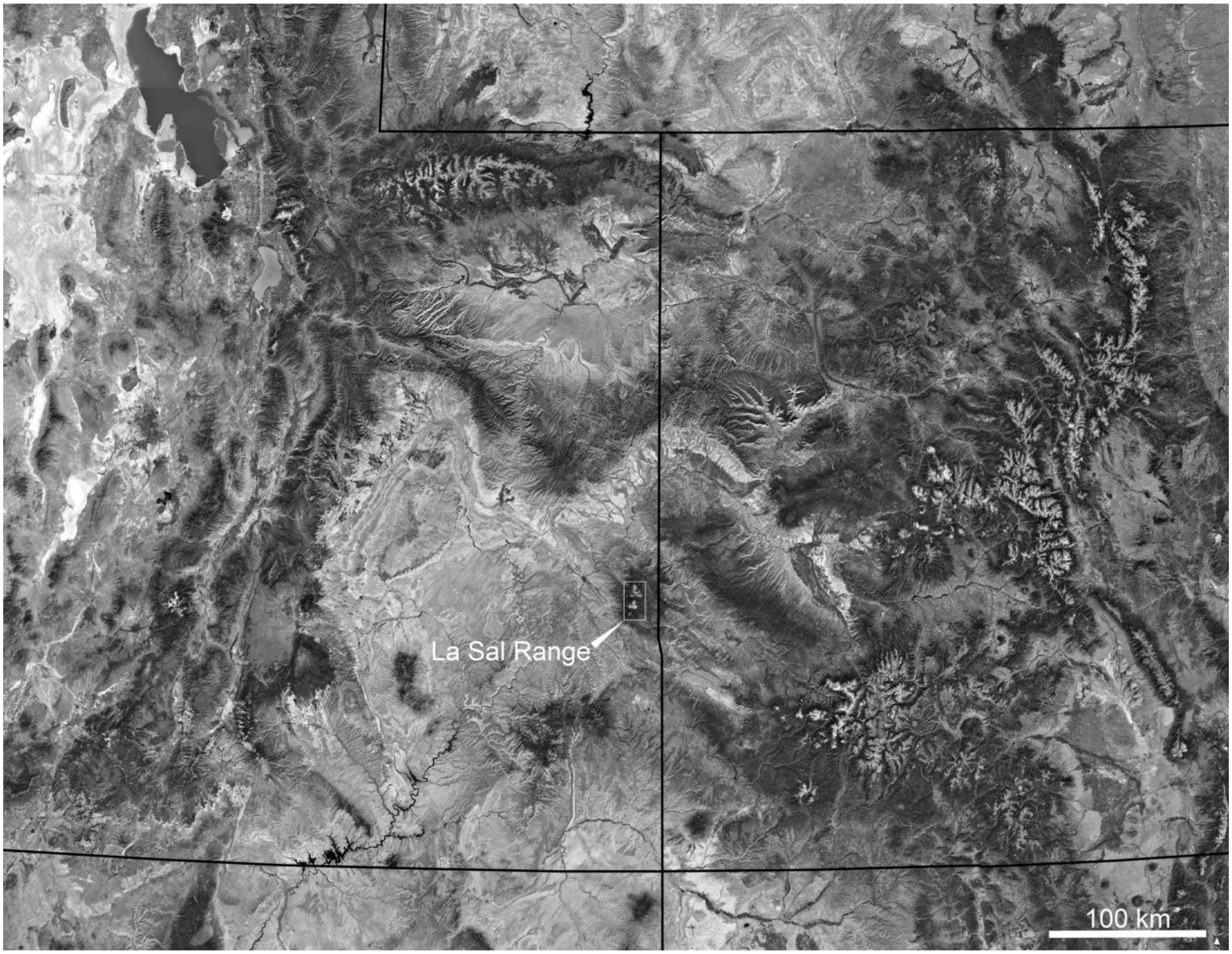

Sampling localities (indicated with black-dotted pattern) in ‘North Group’ of the La Sal Range, Utah, USA.

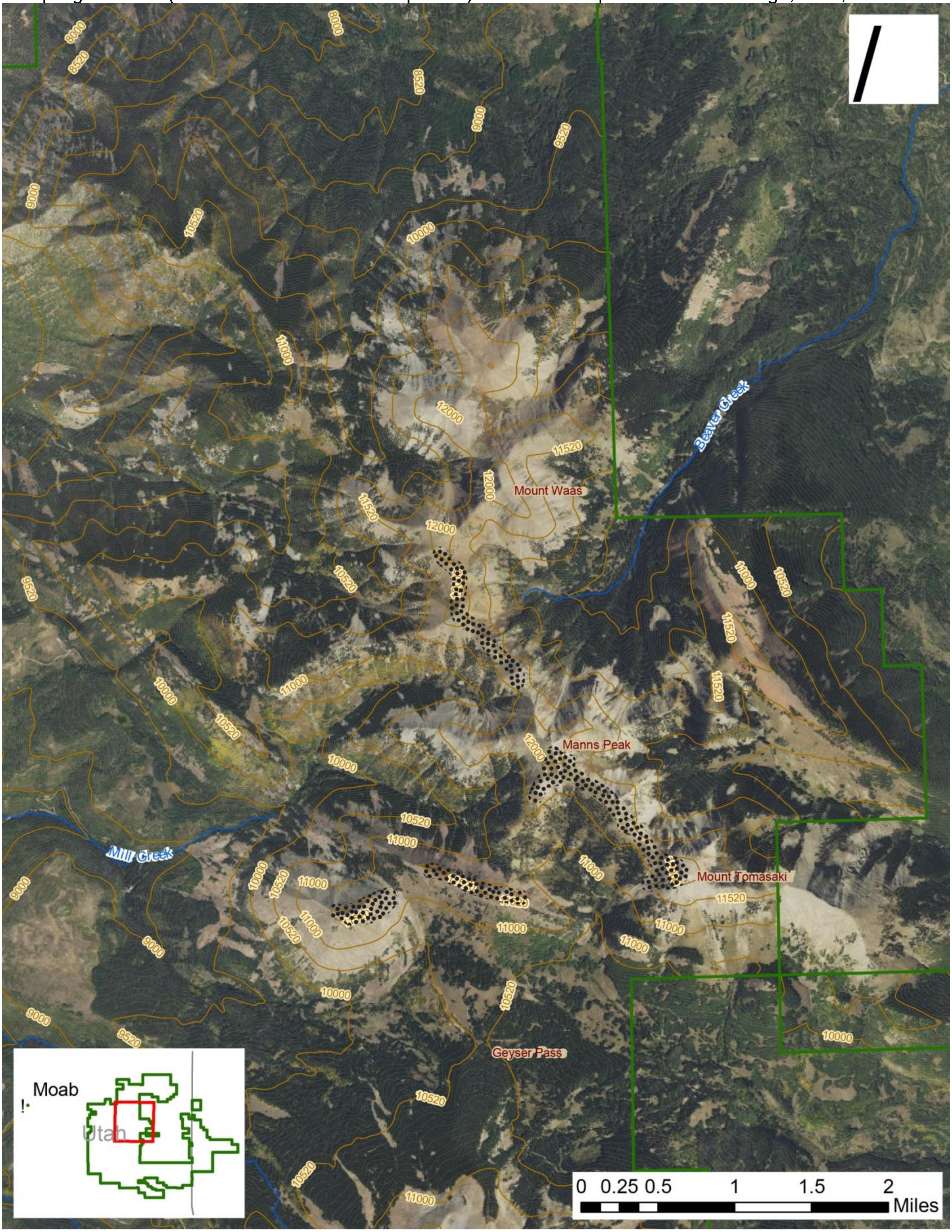

Sampling localities indicated with black-dotted pattern) in 'Souths Group' of the La Sal Range, Utah, USA

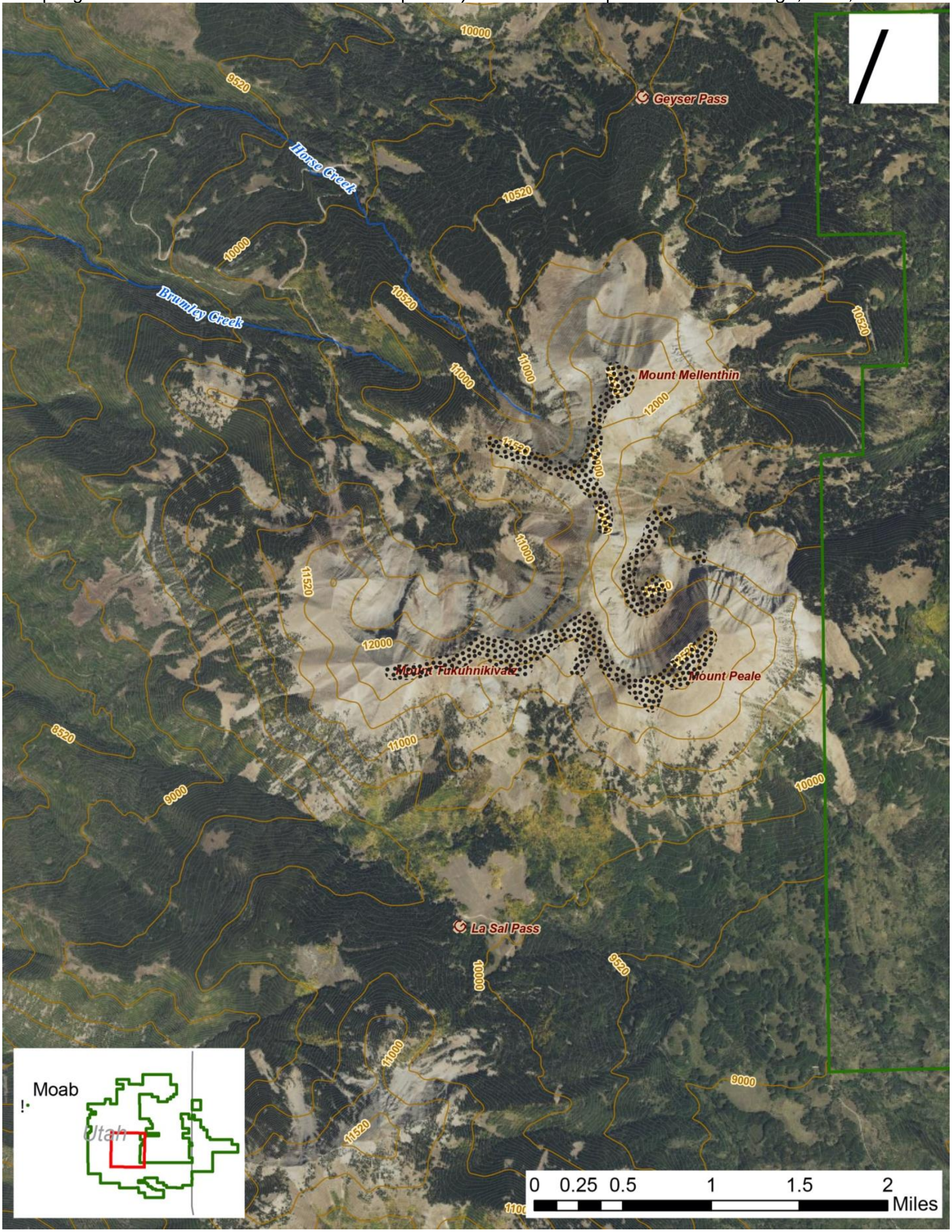

Supplement: Supplementary file 1 — File S1 [file ECE3-11-11090-s002.pdf]
