# Supplementary material for: Alpine lichen diversity in an isolated sky island in the Colorado Plateau, USA—Insight from an integrative biodiversity inventory
Source: Ecol Evol. 2021 Jul 14;11(16):11090–101. doi: 10.1002/ece3.7896 (PMC8366874; doi:10.1002/ece3.7896)

Files S3. Family-level phylogenies inferred from ITS multiple sequence alignments.

Acarosporaceae: 12 candidate species (CS); 11 integrative species (combining morphologically similar CS)

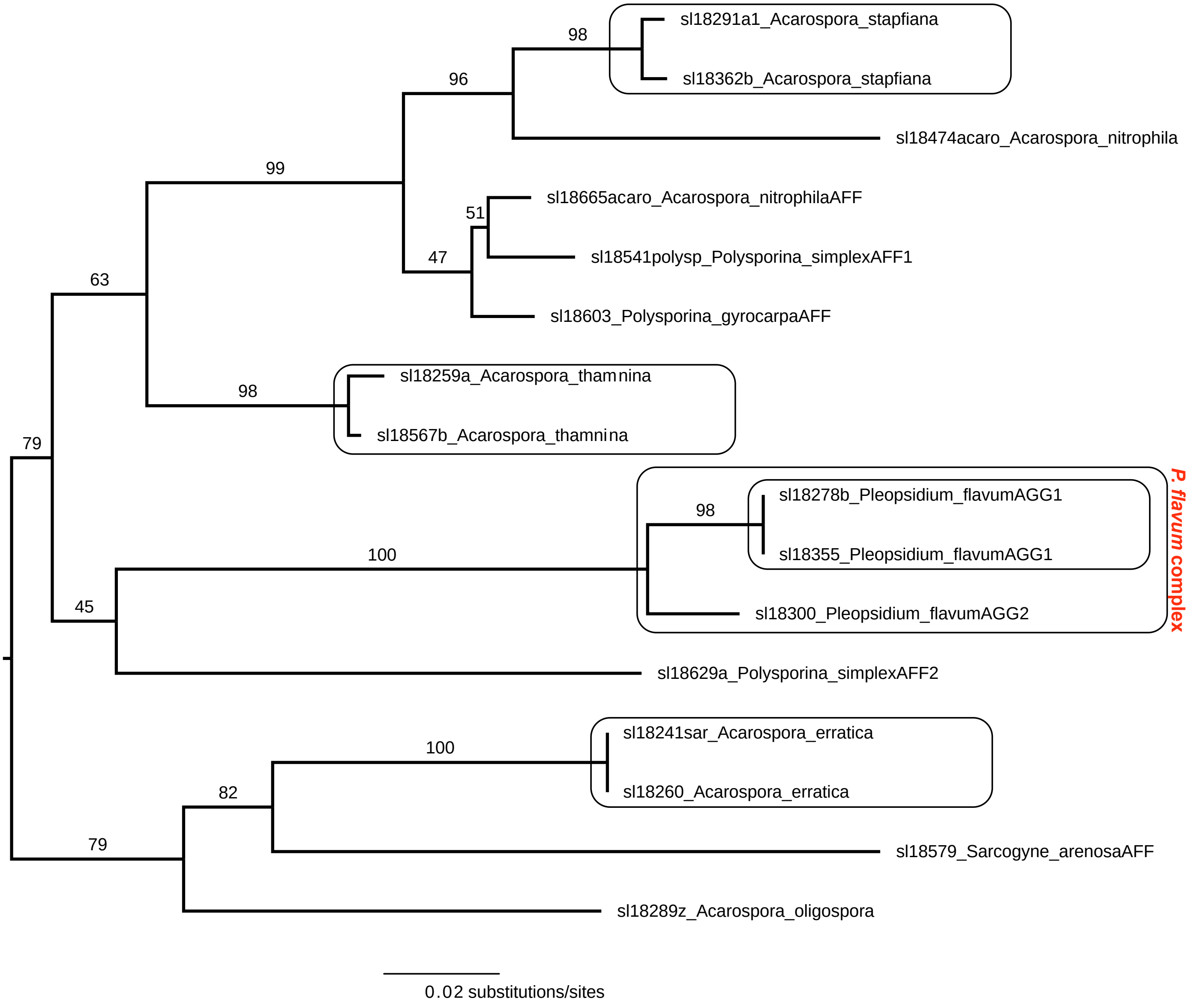

Caliciaceae*: 3 candidate species (CS); 3 integrative species (combining morphologically similar CS)

Candelariaceae: 18 candidate species (CS); 8 integrative species (combining morphologically similar CS)

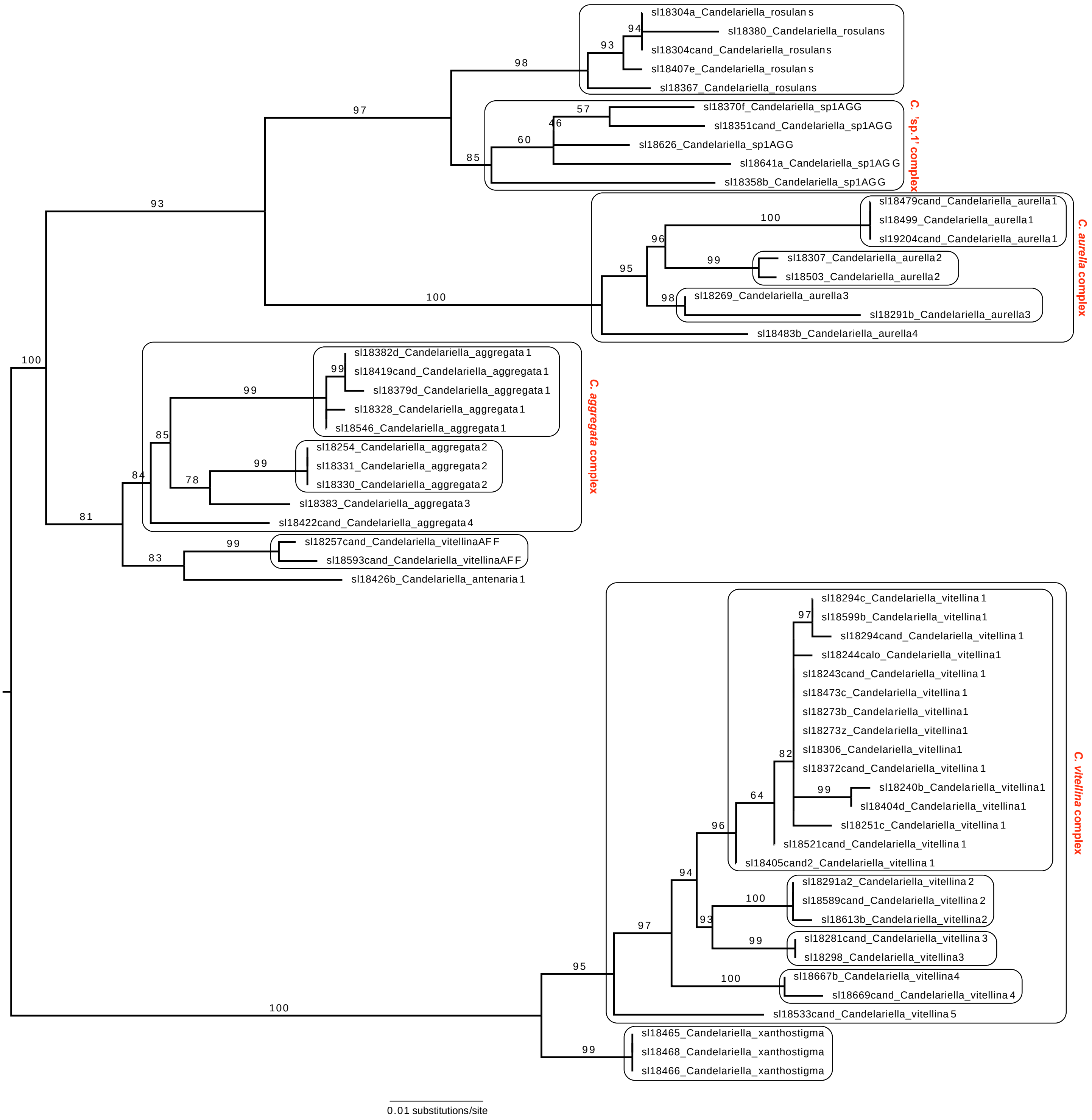

Cladoniaceae: 9 candidate species (CS); 5 integrative species (combining morphologically similar CS)

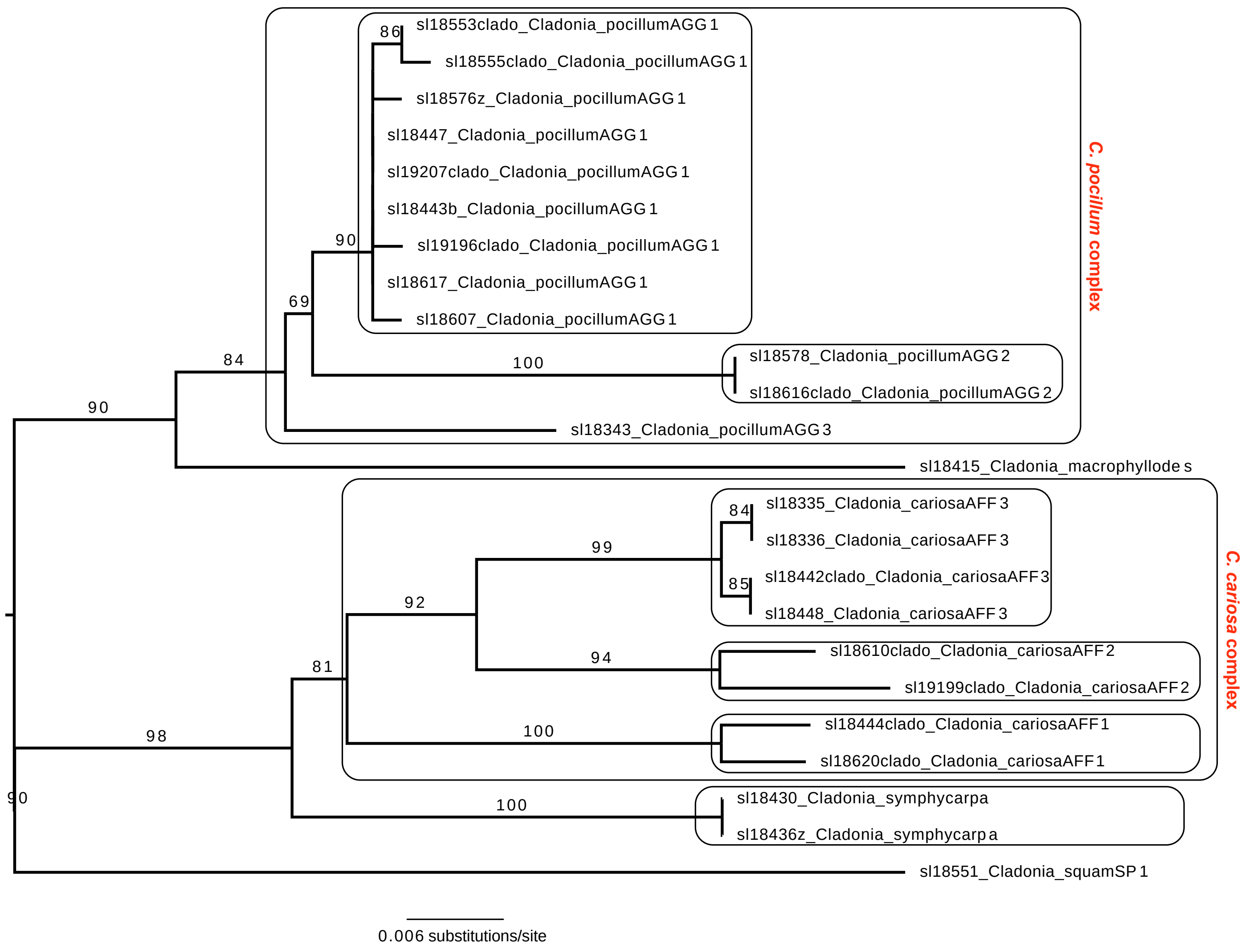

Collemataceae*: 2 candidate species (CS); 2 integrative species (combining morphologically similar CS)

*family represented by only two sequences and phylogeny not inferred. Amplification and sequencing Cyanolichens generally resulted in messy, unusable sequences.

Gyalectaceae*: 1 candidate species (CS); 1 integrative species (combining morphologically similar CS)

*family represented by only one sequence and phylogeny not inferred.

Lecanoraceae: 69 candidate species (CS); 43 integrative species (combining morphologically similar CS)

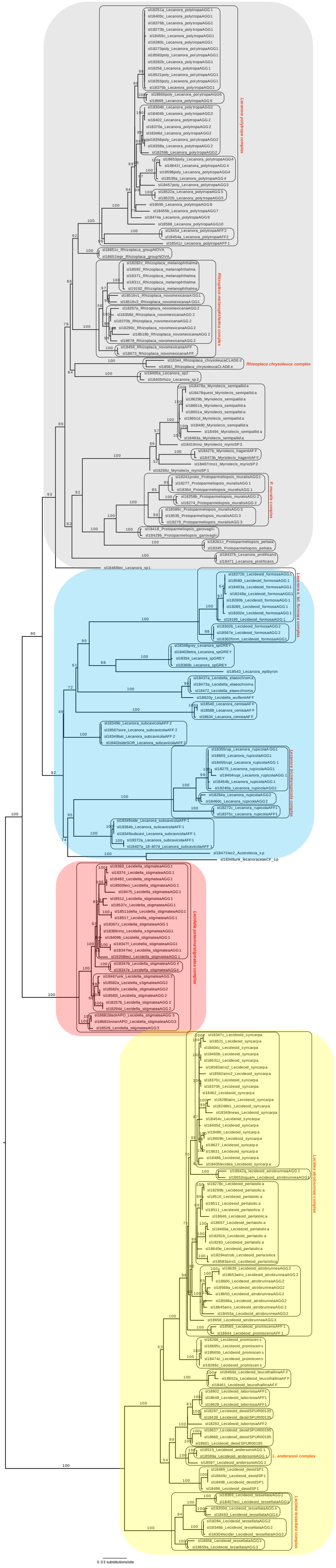

Lecanoraceae: Part 1 (highlighted in grey on right)

32 candidate species (CS); 18 integrative species (combining morphologically similar CS)

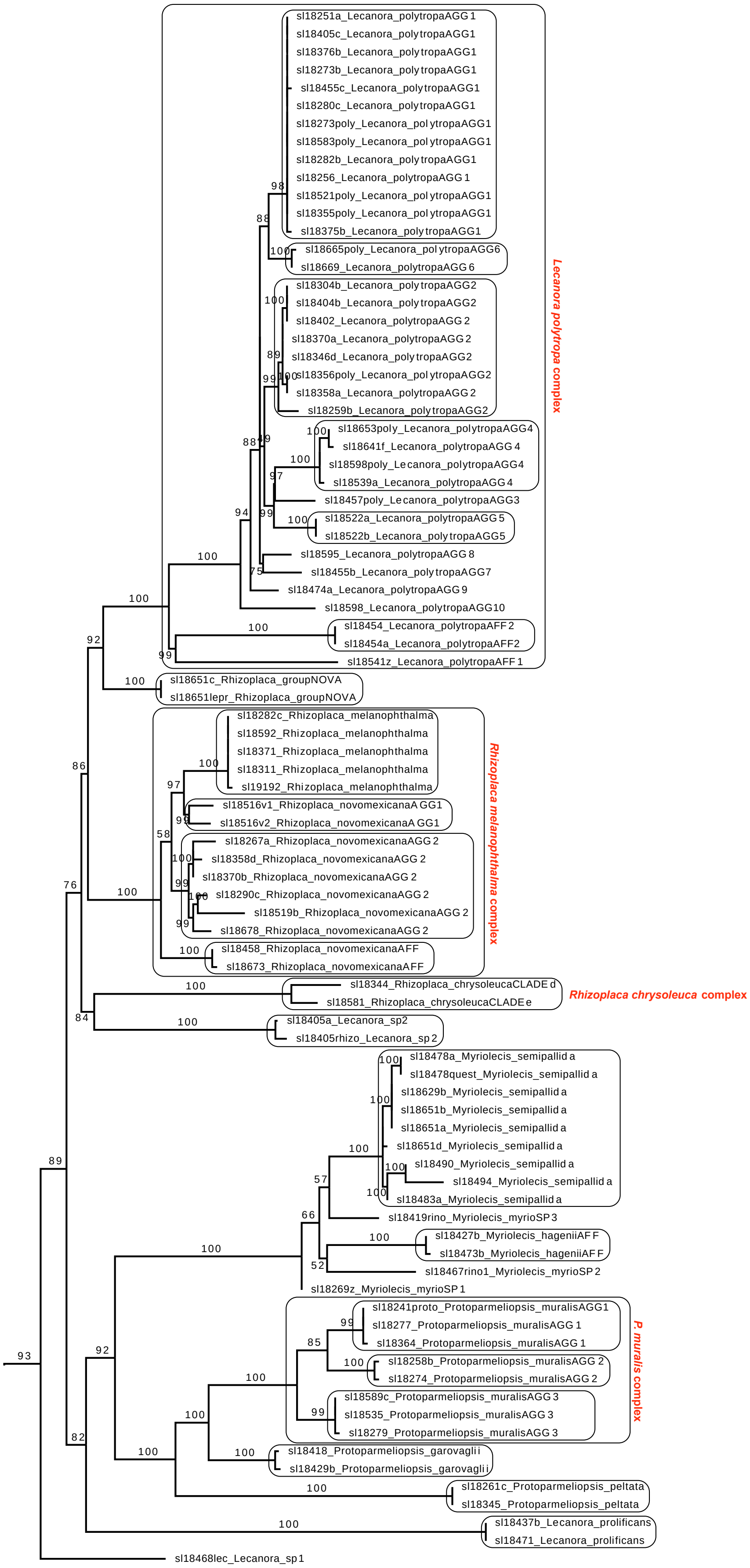

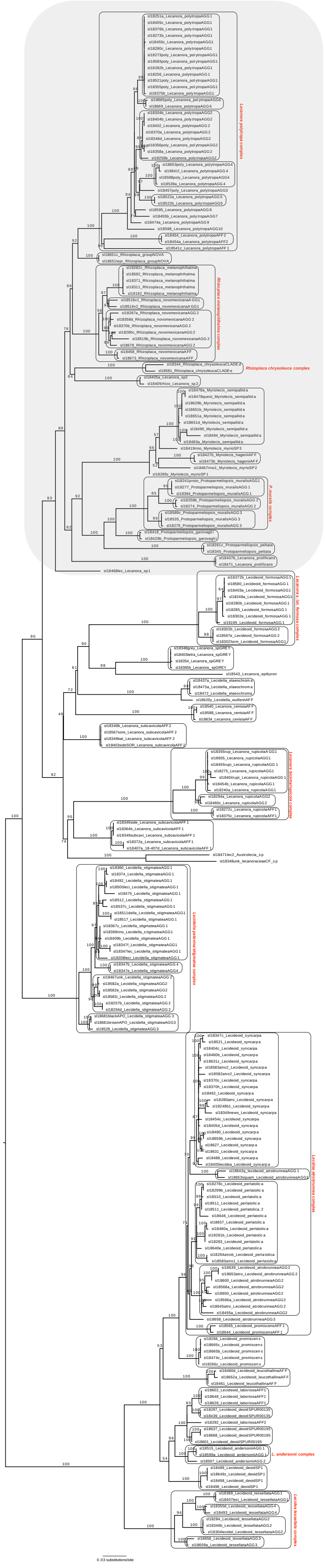

Lecanoraceae: Part 2 (highlighted in blue) & 3 (highlighted in red):

18 candidate species (CS); 12 integrative species (combining morphologically similar CS)

Part 2 – *Lecanora* s. lat. (blue)

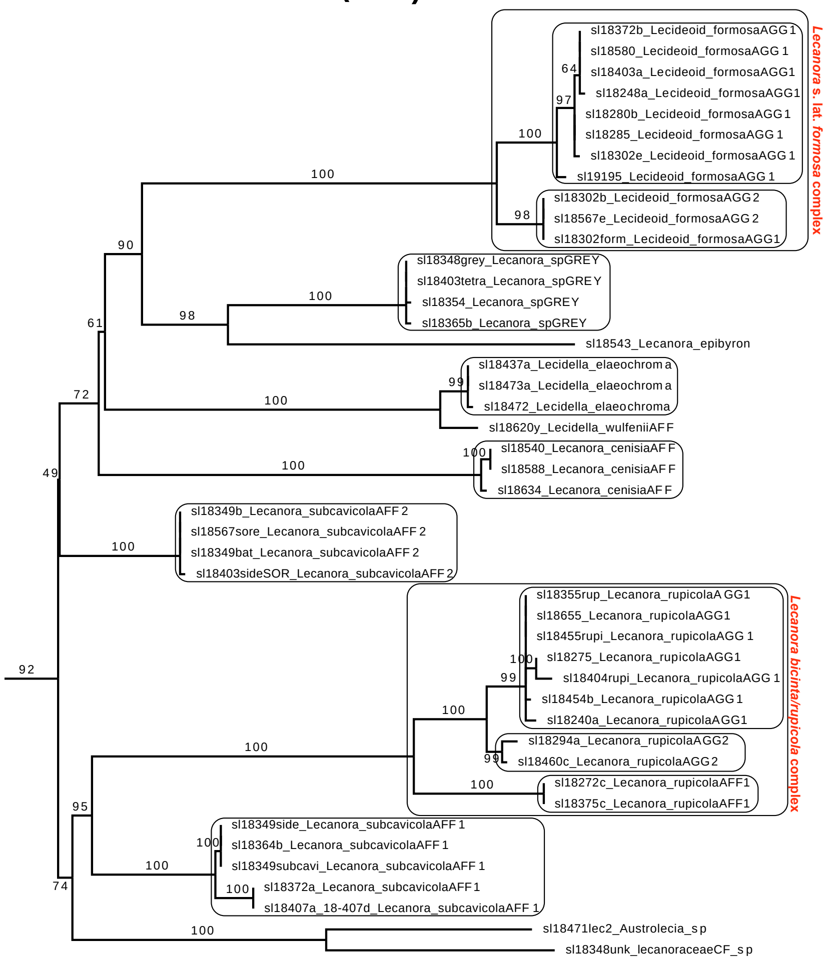

Part 3 – *Lecidella patavine/stigmatea* complex (red)

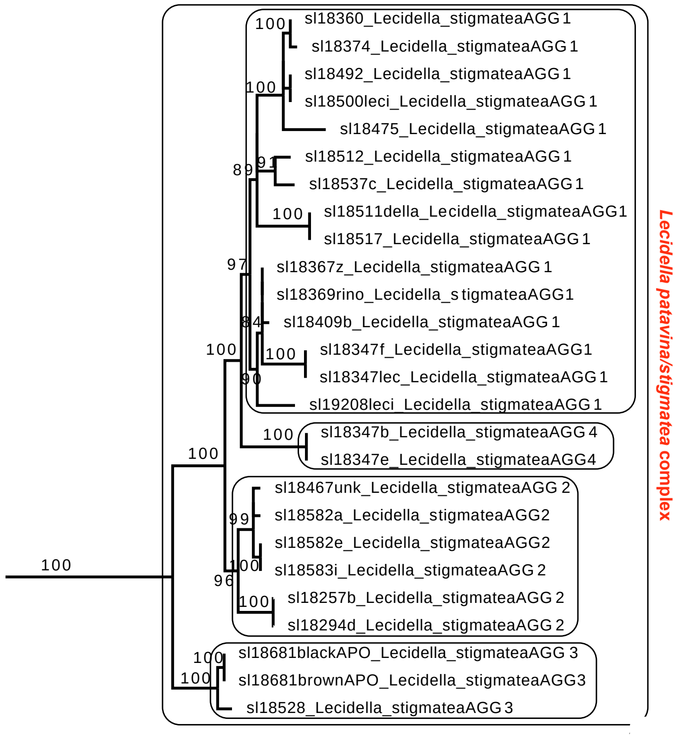

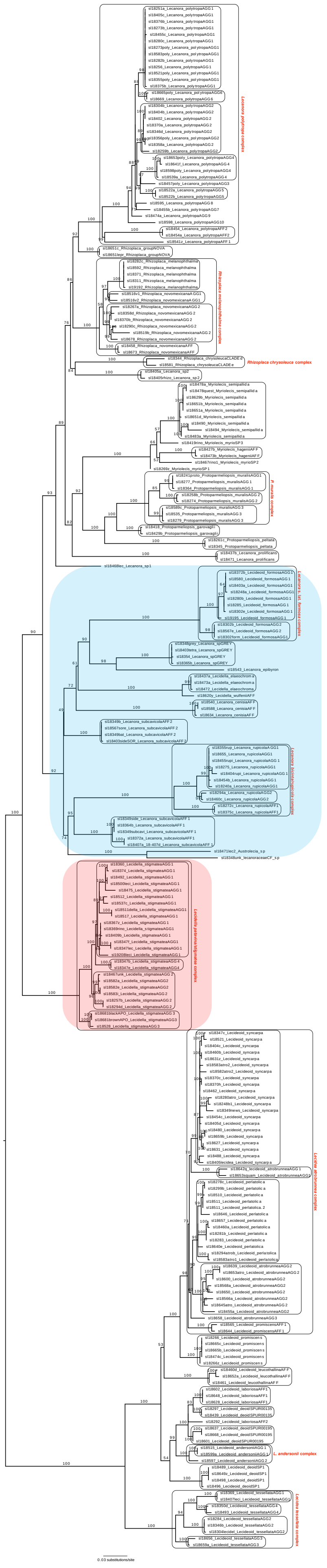

Lecanoraceae: Part 4 – lecideoid Lecanoraceae (highlighted in yellow)

19 candidate species (CS); 13 integrative species (combining morphologically similar CS)

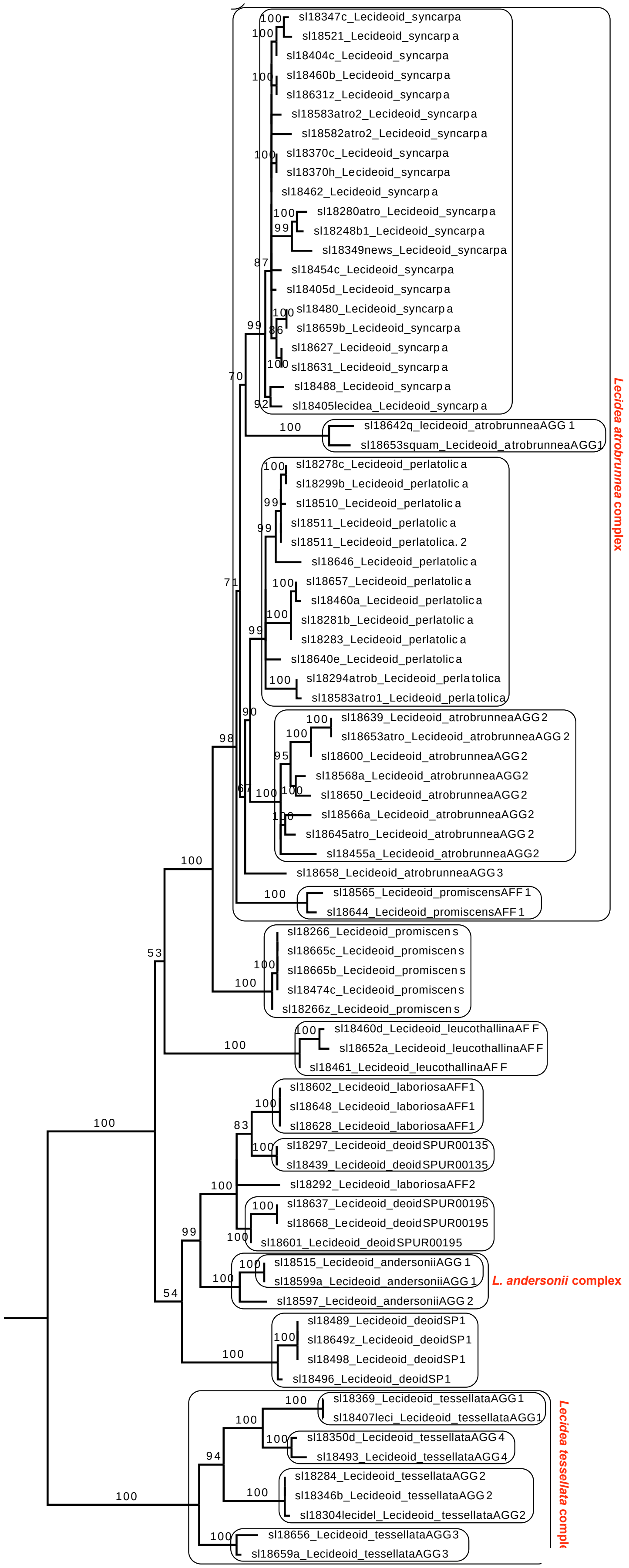

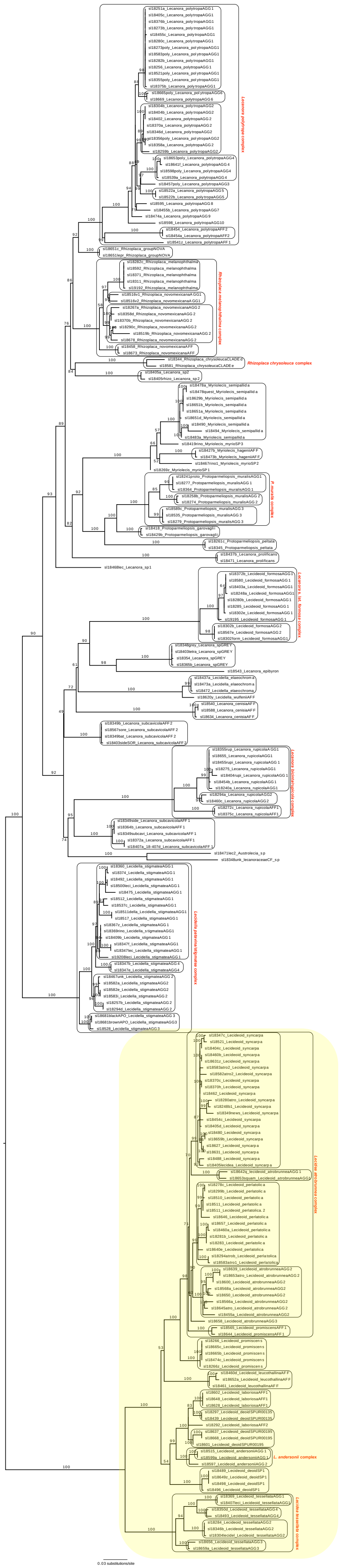

Lecideaceae*: 1 candidate species (CS); 1 integrative species (combining morphologically similar CS)
*family represented by only two sequences and phylogeny not inferred.

Megasporaceae: 22 candidate species (CS); 14 integrative species (combining morphologically similar CS)

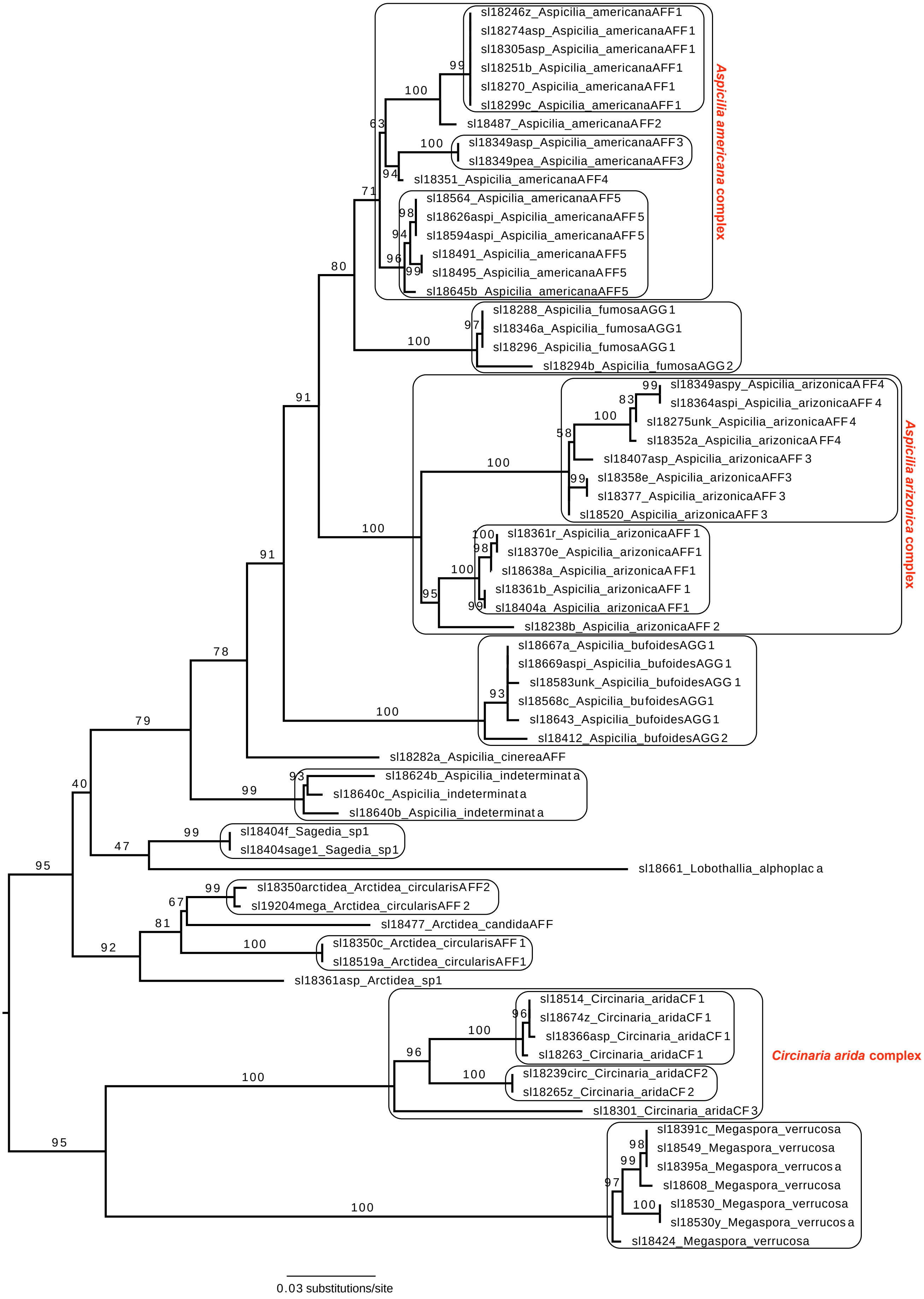

Parmeliaceae: 5 candidate species (CS); 5 integrative species (combining morphologically similar CS)

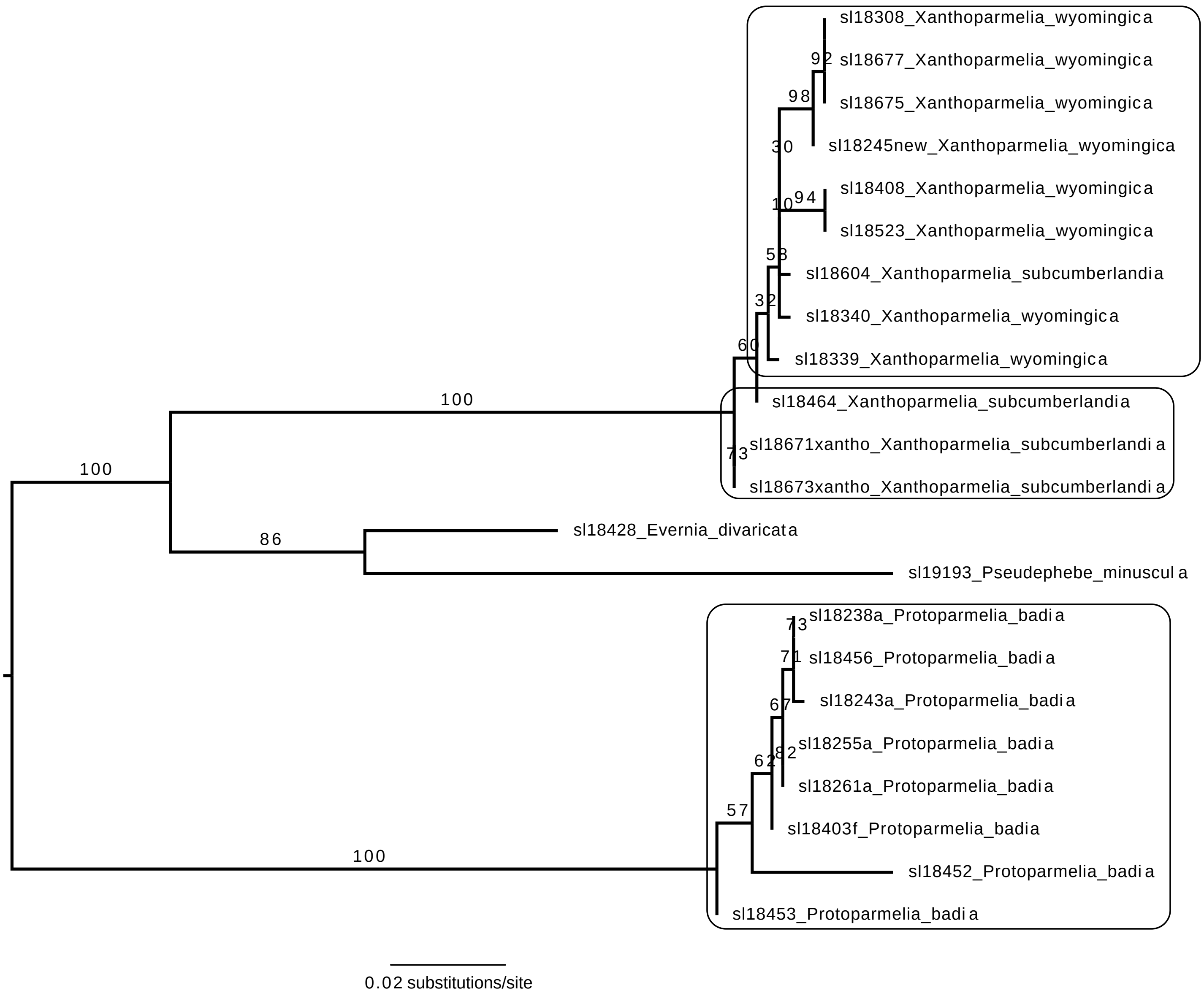

Peltigeraceae: 6 candidate species (CS); 6 integrative species (combining morphologically similar CS)

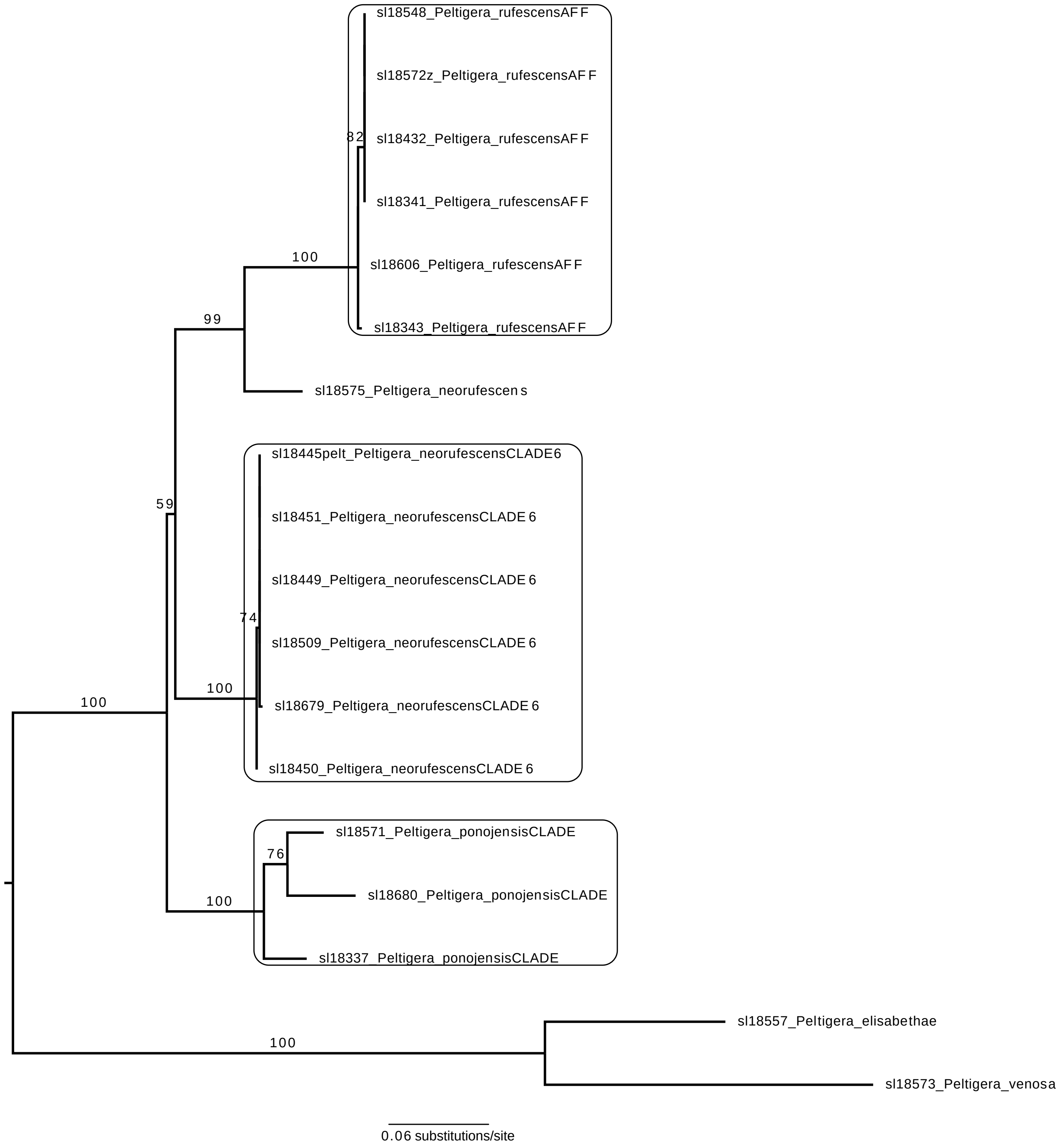

Pertusariaceae*: 1 candidate species (CS); 1 integrative species (combining morphologically similar CS)
*family represented by only three sequences and phylogeny not inferred.

Physciaceae: 18 candidate species (CS); 18 integrative species (combining morphologically similar CS)

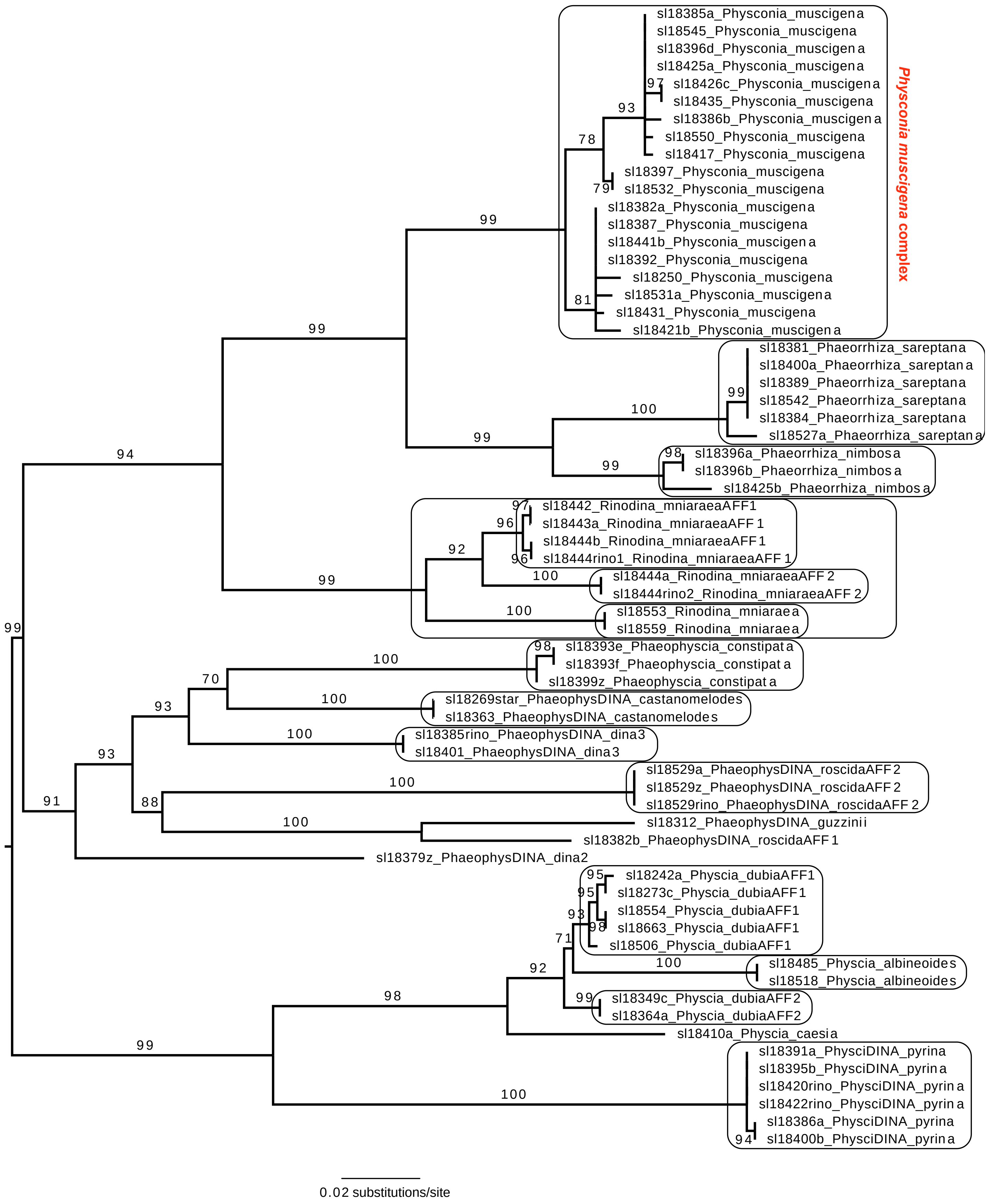

Psoraceae: 3 candidate species (CS); 3 integrative species (combining morphologically similar CS)

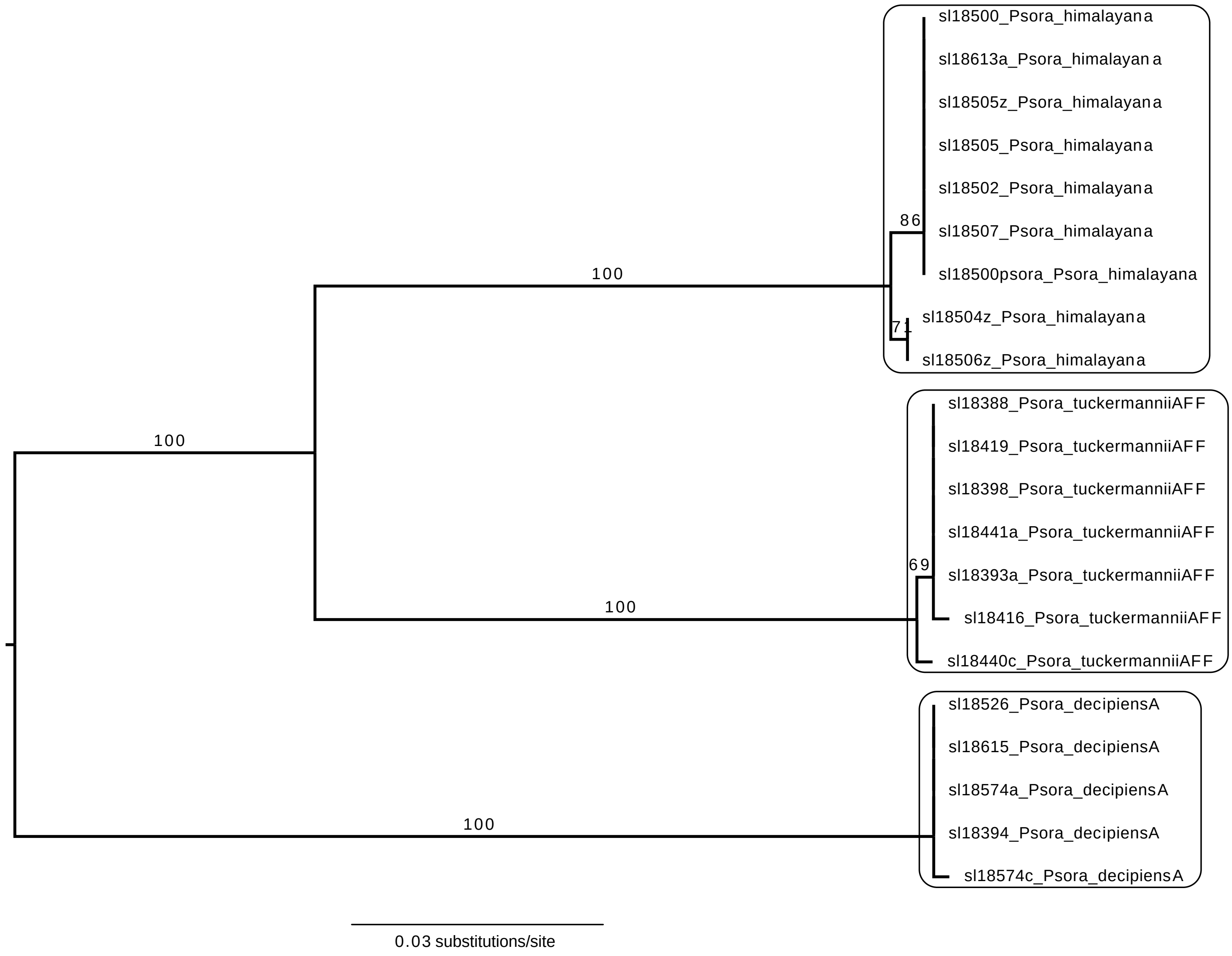

“Pseudoaspicilia”*: 1 candidate species (CS); 1 integrative species (combining morphologically similar CS)

*represented by only a single sequence, phylogeny not inferred

Ramalinaceae*: 1 candidate species (CS); 1 integrative species (combining morphologically similar CS)
*represented by only a single sequence, phylogeny not inferred

Rhizocarpaceae: 6 candidate species (CS); 6 integrative species (combining morphologically similar CS)

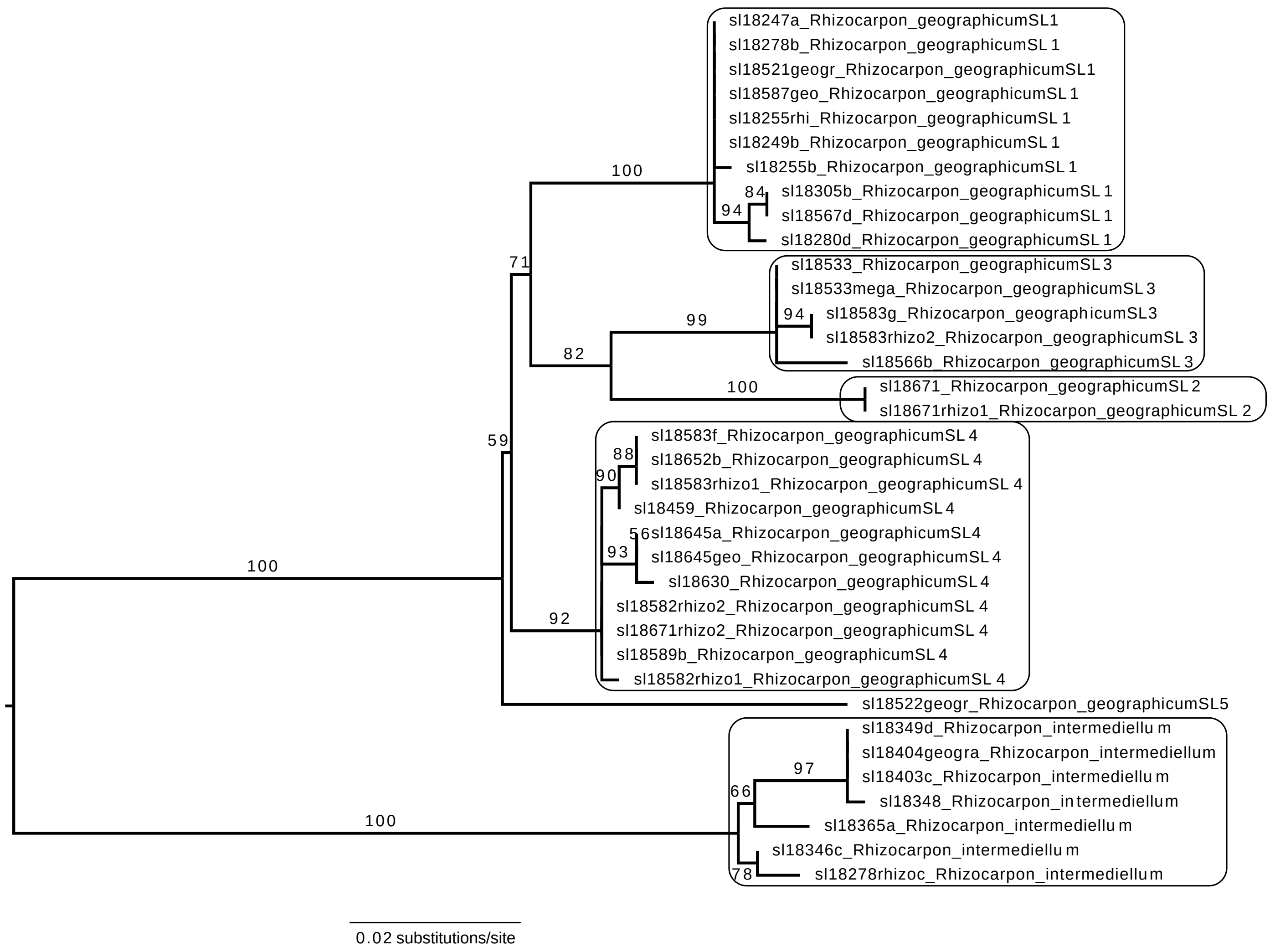

Sporastatiaceae: 1 candidate species (CS); 1 integrative species (combining morphologically similar CS)

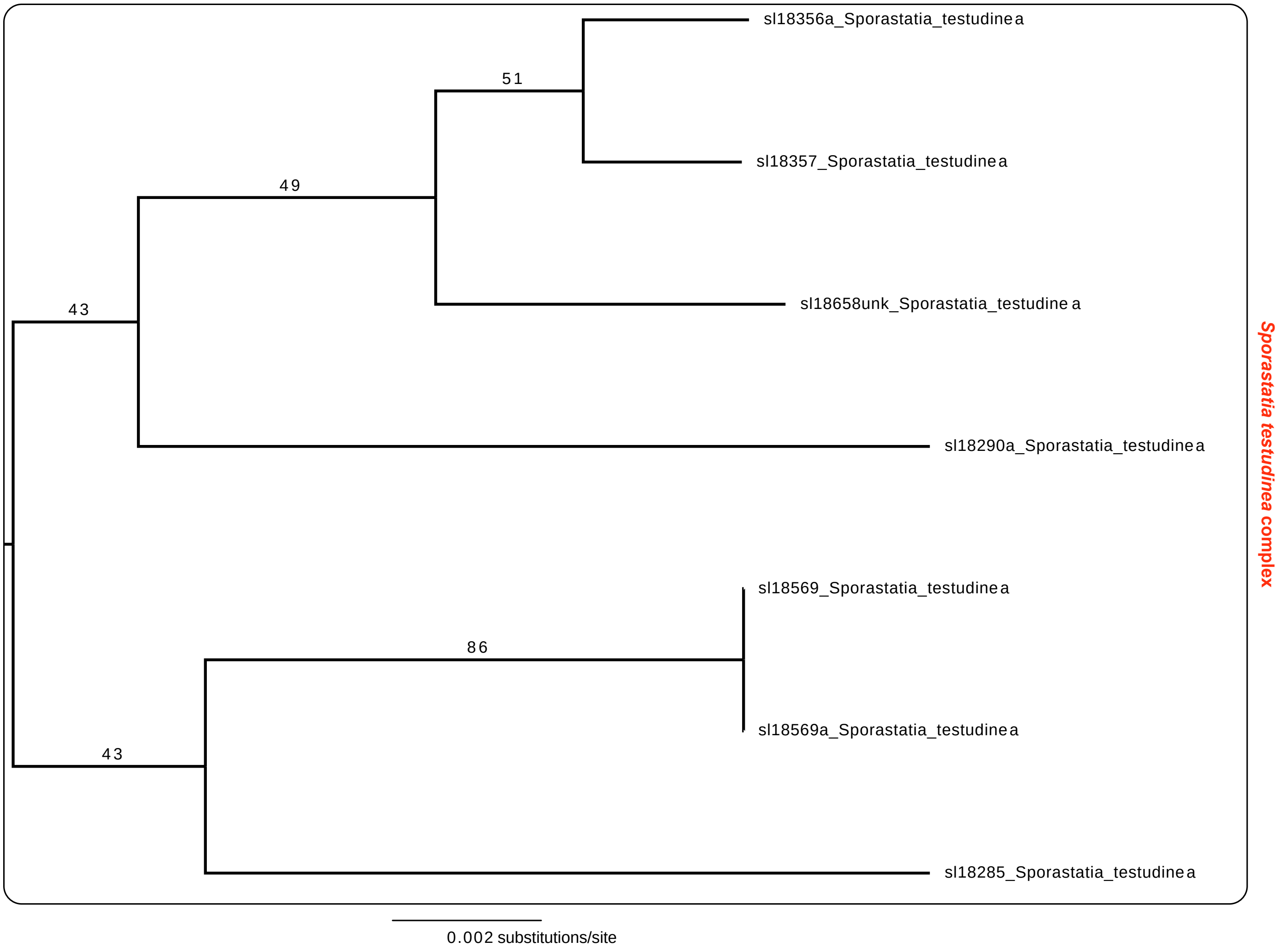

Stereocaulaceae: 4 candidate species (CS); 4 integrative species (combining morphologically similar CS)

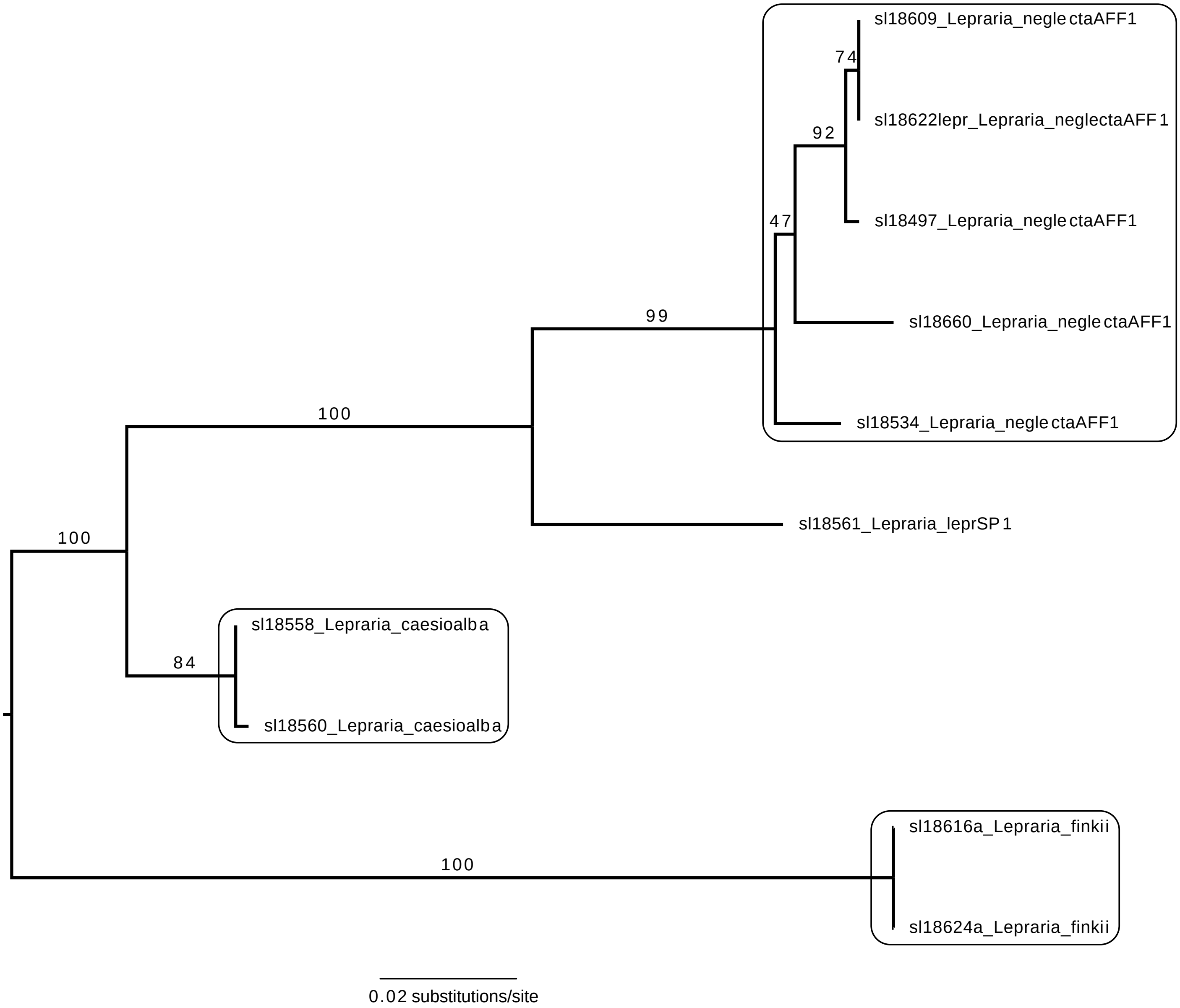

Teloschistaceae: 19 candidate species (CS); 19 integrative species (combining morphologically similar CS)

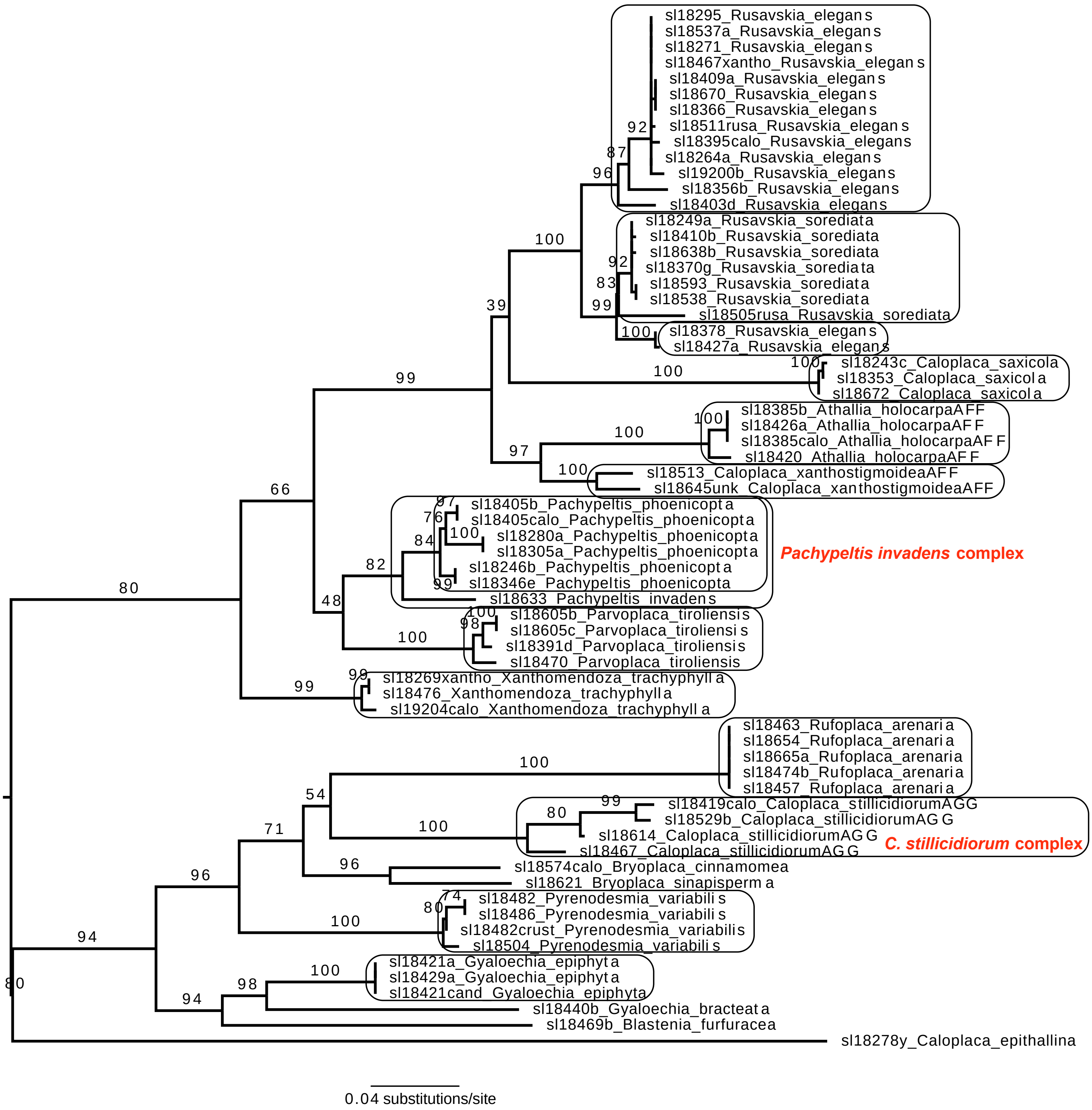

Tephromelataceae*: 2 candidate species (CS); 1 integrative species (combining morphologically similar CS)

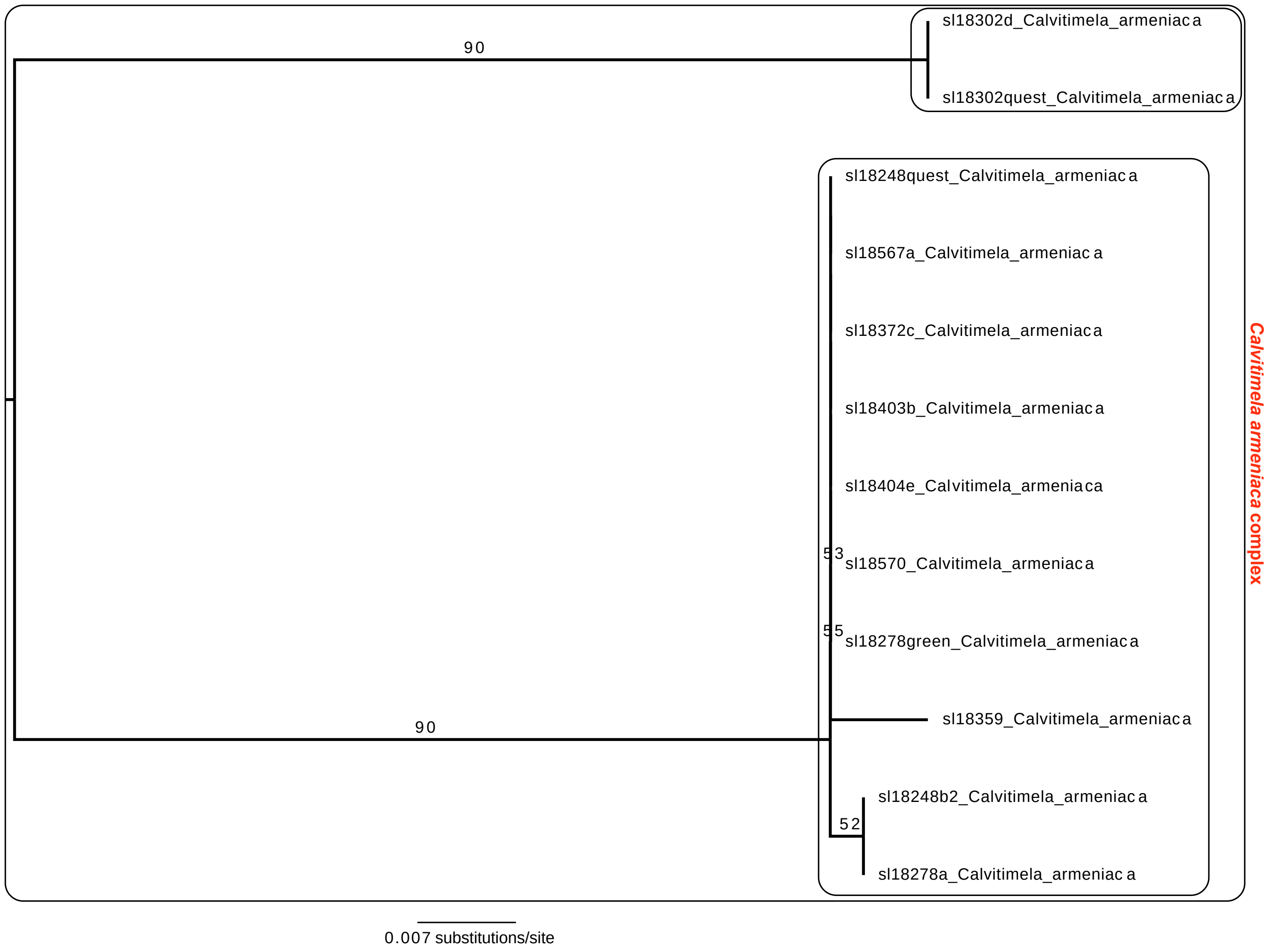

Thelotremataceae*: 1 candidate species (CS); 1 integrative species (combining morphologically similar CS)
**represented by only a single sequence, phylogeny not inferred

Umbilicariaceae: 4 candidate species (CS); 4 integrative species (combining morphologically similar CS)

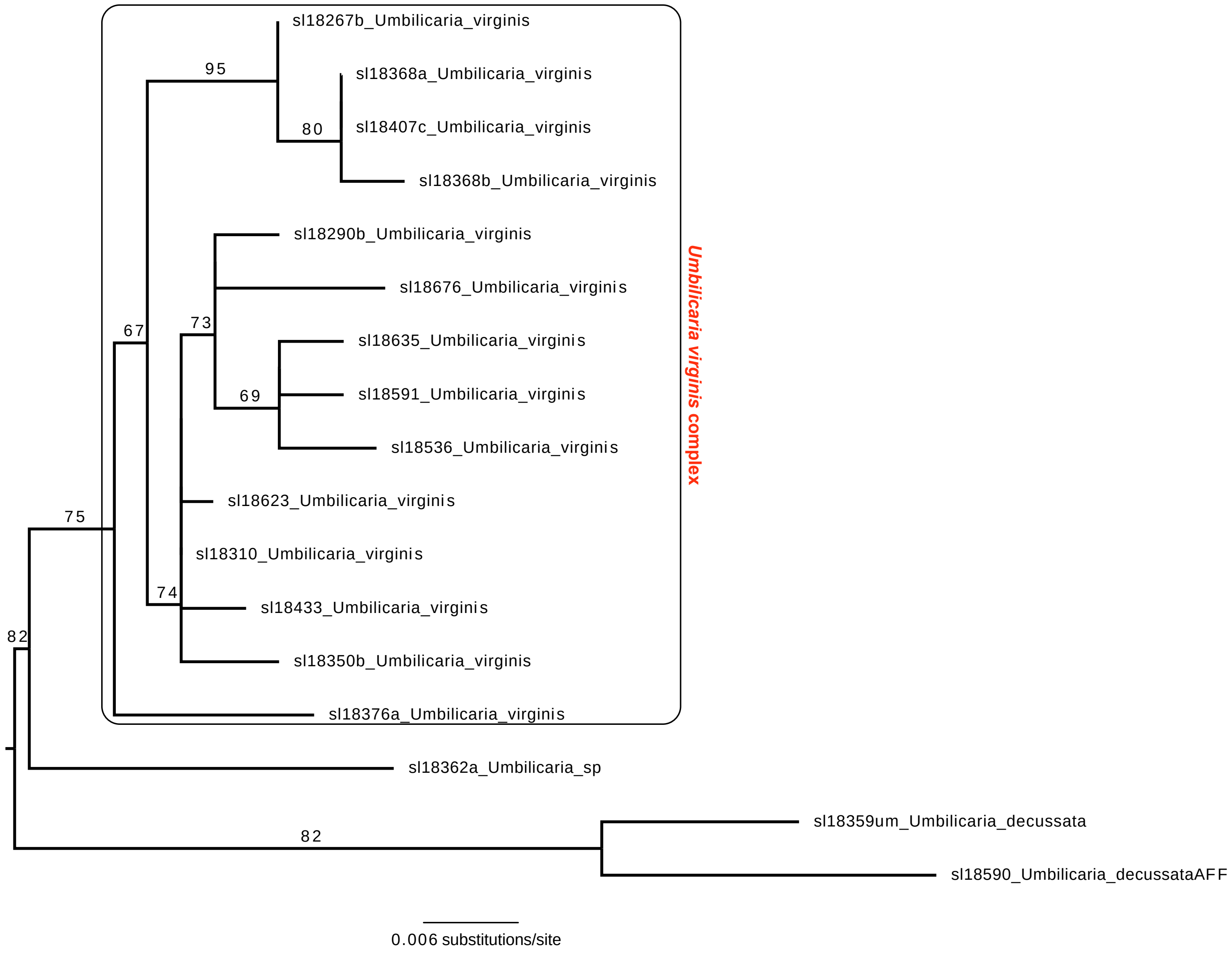

Verrucariaceae: 13 candidate species (CS); 12 integrative species (combining morphologically similar CS)

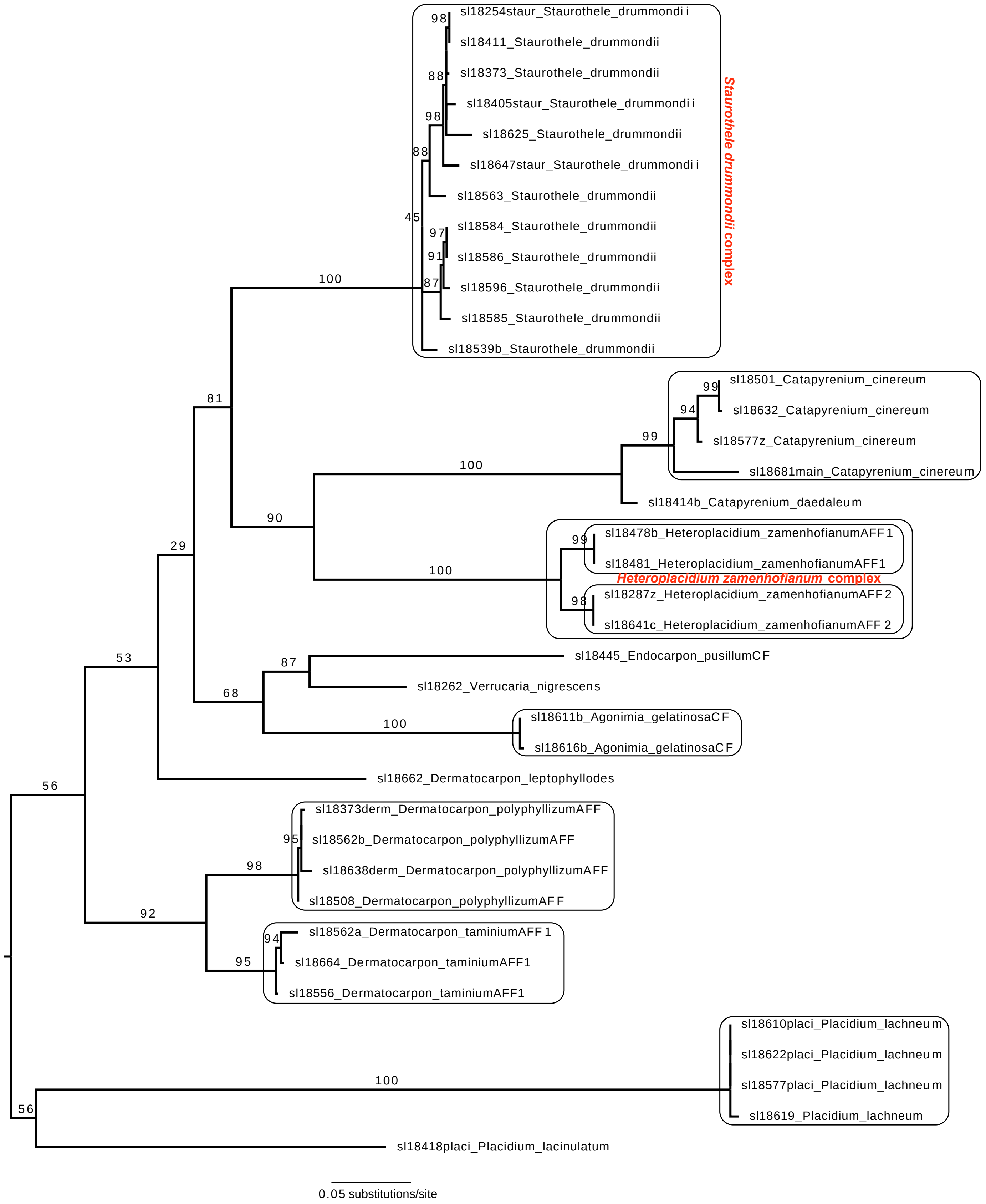

Supplement: Supplementary file 3 — File S3 [file ECE3-11-11090-s005.pdf]
