# Supplementary material for: Alpine lichen diversity in an isolated sky island in the Colorado Plateau, USA—Insight from an integrative biodiversity inventory
Source: Ecol Evol. 2021 Jul 14;11(16):11090–101. doi: 10.1002/ece3.7896 (PMC8366874; doi:10.1002/ece3.7896)
Supplement: Supplementary file 4 — File S4 [file ECE3-11-11090-s006.zip › file_S4_protax_analysis/protax_fungi_results/krona.html]

Javascript must be enabled to view this page.

num
probth


732

732
3

130.765
6

130.764669095559
6

0
1

599.022
3

137.39
6

137.389609968243
6

0
1

1.87306
3

0.470282
6

0.470282030388789
6

0
1

1.40277983202405
3

0
1

14.4143
3

2.74703
6

2.74702856527765
6

0
1

10.9142
3

0.373569
6

0.373568697231465
6

0
1

10.4822
3

0.034758
6

0.0347580477308021
6

0
1

0.0116991
3

0.0116991
6

0
1

0.277518
3

0.277515
6

2.879238284903e-06
3

0
1

0.163875
3

0.153886
6

0.00998879918741857
3

0
1

0.802084
3

0.229944
6

0.479288
3

0.0344664
3

0.0583854729248134
3

0
1

0.254722
3

0.0958334
6

0.158722
3

0.000166084834224284
3

0
1

0.0253887
3

0.0253887
6

0
1

0.0143802
3

0.0143802
6

0
1

4.30931
3

0.178888
6

0.0350218
3

3.02125
3

0.994337
3

0.0798186892436934
3

0
1

0.519315
3

0.5193
6

1.54514171148978e-05
3

0
1

2.55831
3

2.16092
6

0.397319
3

7.44347338493823e-05
3

0
1

0.091594
3

0.0913502
6

0.000243768577009251
3

0
1

0.045177
3

0.0445351
6

0.000641879599165812
3

0
1

1.3740557886613
3

0
1

0.0584402924014764
3

0
1

0.753099073307137
3

0
1

417.086
3

34.8928
6

34.8928047496366
6

0
1

14.1717
3

0.110545
6

0.110545478207301
6

0
1

13.9745
3

0.0219982
6

0.0219981577023236
6

0
1

8.24825
3

8.04895
6

0.124003
3

0.0495426
3

0.0257509275026668
3

0
1

0.136656
3

0.136656
6

0
1

1.13192
3

0.225213
6

0.89145
3

0.0152571454323931
3

0
1

1.78992
3

0.0991545
6

1.69004
3

0.000722547949835617
3

0
1

2.27054
3

2.20952
6

0.060976
3

4.74262738393433e-05
3

0
1

0.375221233627522
3

0
1

0.0866538130184402
3

0
1

27.1172
3

0.994356
6

0.994355869863565
6

0
1

0.462843
3

0.055158
3

0.055158
6

0
1

0.407684735041106
3

0
1

25.3163
3

1.79337
3

1.79337
6

0
1

0.401405
3

0.401405
6

0
1

3.71243
3

2.90267
6

0.0331377
3

0.76413
3

0.0124937750346249
3

0
1

5.55519
3

2.19745
6

0.0413794
3

0.345577
3

0.952467
3

0.211425
3

1.72357
3

0.0833119340017374
3

0
1

8.37503
3

1.43444
6

6.91303
3

0.0275609567988226
3

0
1

4.65256
3

4.65256
6

0
1

0.82627683250709
3

0
1

0.343778354029549
3

0
1

53.5672
3

0.0806341
6

0.0806341462460516
6

0
1

53.0017
3

0.017249
6

0.0172490191153463
6

0
1

52.773
3

52.773
6

0
1

0.211488338218793
3

0
1

0.0412896
3

0.0254039
3

0.0253914
6

1.24879801305898e-05
3

0
1

0.0158856450234688
3

0
1

0.443531320329621
3

0
1

165.654
3

4.99842
6

4.99842482945431
6

0
1

19.8582
3

19.5699
3

0.394157
6

0.137349
3

0.12588
3

6.0163
3

0.151455
3

3.64743
3

0.030951
3

0.425576
3

3.36271
3

1.7446
3

0.209624
3

0.536843
3

2.46613
3

0.320942153674487
3

0
1

0.288215714827889
3

0
1

48.476
3

31.6466
3

17.5924
6

0.641656
3

0.198802
3

2.60181
3

9.7245
3

0.16195
3

0.64657
3

0.0788941403638524
3

0
1

7.3152
3

6.34124
6

0.156811
3

0.817132
3

1.48539883075838e-05
3

0
1

0.0955672
3

0.0955666
6

6.04716553317952e-07
3

0
1

4.07525
3

2.1498
6

1.91258
3

0.0128731197147611
3

0
1

1.76674
3

1.39114
6

0.373897
3

0.00169968268478043
3

0
1

3.57666315116163
3

0
1

0.38624
3

0.192611
3

0.19254
6

7.08693271536109e-05
3

0
1

0.19362941937664
3

0
1

51.6263
3

0.366343
3

0.366343
6

0
1

0.357604
3

0.308124
6

0.0410574
3

0.00842292163551873
3

0
1

0.045859
3

0.045859
6

0
1

1.08802
3

1.05044
6

0.0113923
3

0.011793
3

0.011793
3

0.00260695082157083
3

0
1

0.0610114
3

0.0609763
6

3.50284628549899e-05
3

0
1

0.442919
3

0.310701
6

0.127898
3

0.00431919172431525
3

0
1

0.503731
3

0.477893
6

0.0179192
3

0.00791867019110204
3

0
1

2.1073
3

2.06622
6

0.0106107
3

0.0162904
3

0.0103878
3

0.00379102231016246
3

0
1

0.0141401
3

0.0141401
6

0
1

0.0388515
3

0.0124628
6

0.0183177
3

0.00807111490817784
3

0
1

0.299922
3

0.278271
6

0.0185338
3

0.00311737871425748
3

0
1

0.0110547
3

0.0110547
6

0
1

0.125858
3

0.125857
6

1.68346671131081e-07
3

0
1

0.11104
3

0.0809364
6

0.0209016
3

0.00920243329084235
3

0
1

1.11751
3

1.10274
6

0.0118462
3

0.00291732632741232
3

0
1

3.05531
3

3.05529
6

1.14373474593421e-05
3

0
1

0.174481
3

0.174371
6

0.000110530171755274
3

0
1

2.02854
3

0.471983
6

1.55653
3

2.11349883203482e-05
3

0
1

0.208939
3

0.208626
6

0.00031225153455966
3

0
1

2.8433
3

2.81165
6

0.0242771
3

0.00737777545159352
3

0
1

0.166924
3

0.162261
6

0.00466264171188344
3

0
1

3.16546
3

3.1649
6

0.000559121597017764
3

0
1

0.0410923
3

0.0361608
6

0.00493144651619172
3

0
1

10.6979
3

3.66399
6

5.57365
3

1.28855
3

0.17175041652843
3

0
1

22.5531190595119
3

0
1

12.0954
3

0.0304922
3

0.0304423
6

4.99663017616316e-05
3

0
1

11.2273
3

10.7745
6

0.305952
3

0.146878947939905
3

0
1

0.837604509612087
3

0
1

3.94063
3

0.0119422
6

0.0119421633779159
6

0
1

0.010789
3

0.0107887
6

3.23664556706529e-07
3

0
1

0.0278014
3

0.0278014
6

0
1

0.370638
3

0.037125
6

0.333513
3

0
1

0.116087
3

0.116076
6

1.0985564852739e-05
3

0
1

0.0981534
3

0.0981526
6

8.08893733106331e-07
3

0
1

3.30522001780963
3

0
1

0.0612346
3

0.0612346393567994
3

0
1

6.67327
3

0.1501
6

0.150100491157431
6

0
1

5.34047
3

2.56283
6

0.490282
3

0.193152
3

2.08491
3

0.00929888333744211
3

0
1

0.687194
3

0.687194
6

0
1

0.495506241056974
3

0
1

1.76109
3

0.24626
6

0.246260089876805
6

0
1

0.54414
3

0.544136
3

4.45494708933847e-06
3

0
1

0.278912
3

0.278912
6

0
1

0.691776541321566
3

0
1

15.7770278649587
3

0
1

31.8781
3

1.03505
6

1.03505206731028
6

0
1

30.5251
3

30.0749
3

30.0326
6

0.0423079755278479
3

0
1

0.450178438624285
3

0
1

0.317950808110837
3

0
1

0.906103
3

0.892336
3

0.0320157
6

0.032015663870287
6

0
1

0.845427
3

0.344718
3

0.50071
3

2.77254133984606e-07
3

0
1

0.0148925381635205
3

0
1

0.0137678608867468
3

0
1

9.056
3

0.565482
6

0.565481577746554
6

0
1

0.141571
3

0.141571243820622
3

0
1

0.0621956
3

0.0621956290587688
3

0
1

7.83877
3

0.156499
6

0.15649933821316
6

0
1

7.64281
3

7.64265
6

0.000162241181717704
3

0
1

0.0394613468796283
3

0
1

0.447986468992069
3

0
1

6.04201
3

0.262319
6

0.262318805029947
6

0
1

4.23223
3

1.2676
3

0.963884
6

0.272514
3

0.0311982027913544
3

0
1

2.61089
3

1.90402
6

0.668163
3

0.0387108088076978
3

0
1

0.0202537
3

0.0202409
3

1.27557842567252e-05
3

0
1

0.333489097488956
3

0
1

0.694823
3

0.664095
3

0.655028
6

0.00906651534362035
3

0
1

0.0307277855571111
3

0
1

0.852639413187253
3

0
1

3.50275
3

3.35824
3

3.30791
3

0.163612
6

3.07968
3

0.0132277
3

0.0513873132215911
3

0
1

0.0503253673488868
3

0
1

0.14450762414099
3

0
1

34.9734
3

1.28663
6

1.2866278217518
6

0
1

32.6471
3

0.34502
6

0.345020074565997
6

0
1

4.62563
3

3.24538
6

1.26421
3

0.0344896
3

0.0815529024318338
3

0
1

3.63643
3

1.7896
6

1.84566
3

0.00117018917282641
3

0
1

13.6922
3

9.26881
6

0.807545
3

0.253213
3

0.208139
3

3.11751
3

0.0369835008991113
3

0
1

1.64671
3

1.6467
6

1.34329721708237e-05
3

0
1

0.0457407
3

0.0457402
6

4.57404674546191e-07
3

0
1

0.341255
3

0.272256
6

0.043266
3

0.0168798
3

0.00885348421956046
3

0
1

0.128072
3

0.127933
6

0.000139707407335682
3

0
1

2.66577
3

2.66577
6

6.08990663186759e-06
3

0
1

0.497249
3

0.495766
6

0.00148255927674329
3

0
1

0.0653166
3

0.0653165
6

1.01828026136119e-07
3

0
1

0.743694
3

0.74368
6

1.42555865587646e-05
3

0
1

0.0326382
3

0.0326382
6

0
1

0.373279
3

0.17345
6

0.197076
3

0.00275278046831723
3

0
1

3.80804739724661
3

0
1

1.03968316804686
3

0
1

16.2009
3

0.390079
6

0.390078635858133
6

0
1

0.416194
3

0.0233923
3

0.0147175
6

0.00867479106847141
3

0
1

0.212165
3

0.212164
6

8.43972996406039e-07
3

0
1

0.180637237385344
3

0
1

14.6851
3

0.804454
3

0.394474
6

0.0769371
3

0.252252
3

0.0807905179587253
3

0
1

13.252
3

0.458705
6

2.43381
3

0.0961884
3

0.384527
3

9.67338
3

0.205424340242018
3

0
1

0.439762
3

0.0911239
6

0.295943
3

0.0526943583797083
3

0
1

0.188896655573636
3

0
1

0.70949775079816
3

0
1

0.103731
3

0.0107203
6

0.0107202586053184
6

0
1

0.0453809
3

0.0301551
3

0.0256285
6

0.0045266298611835
3

0
1

0.0152257522377163
3

0
1

0.0476300143604745
3

0
1

19.0205461762806
3

0
1

1.9311
3

0.761342
6

0.761341551303947
6

0
1

0.168568
3

0.0554907
3

0.0291838
3

0.0291777
6

6.12796384562297e-06
3

0
1

0.0263068468061093
3

0
1

0.0474509
3

0.0126099
3

0.0126099
6

0
1

0.034840951305236
3

0
1

0.0656260415581372
3

0
1

1.00119575702391
3

0
1

1.79198
3

0.0435042
6

0.0435042193371407
6

0
1

1.74847088441556
3

0
1

24.5351315091318
3

0
1

2.21382669221305
3

0
1
